# Cancer evolution: Darwin and beyond

**DOI:** 10.15252/embj.2021108389

**Published:** 2021-08-30

**Authors:** Roberto Vendramin, Kevin Litchfield, Charles Swanton

**Affiliations:** ^1^ Cancer Research UK Lung Cancer Centre of Excellence University College London Cancer Institute London UK; ^2^ Cancer Evolution and Genome Instability Laboratory The Francis Crick Institute London UK

**Keywords:** cancer, cancer evolution, cancer therapy, tumour heterogeneity, Cancer

## Abstract

Clinical and laboratory studies over recent decades have established branched evolution as a feature of cancer. However, while grounded in somatic selection, several lines of evidence suggest a Darwinian model alone is insufficient to fully explain cancer evolution. First, the role of macroevolutionary events in tumour initiation and progression contradicts Darwin's central thesis of gradualism. Whole‐genome doubling, chromosomal chromoplexy and chromothripsis represent examples of single catastrophic events which can drive tumour evolution. Second, neutral evolution can play a role in some tumours, indicating that selection is not always driving evolution. Third, increasing appreciation of the role of the ageing soma has led to recent generalised theories of age‐dependent carcinogenesis. Here, we review these concepts and others, which collectively argue for a model of cancer evolution which extends beyond Darwin. We also highlight clinical opportunities which can be grasped through targeting cancer vulnerabilities arising from non‐Darwinian patterns of evolution.

## Graphical abstract

Evolutionary concepts explaining cancer progression, the selective pressures acting on tumours, and their therapeutic implications are discussed in this comprehensive review.

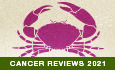

## Introduction

In his revolutionary work (Darwin, 1859), Darwin provided an evolutionary framework which enabled the understanding of somatic selection, diversification and extinction through the application of three key concepts: variation, heredity and selection. More than a 100 years later, the observation of heterogeneity in advanced malignancies led Peter Nowell to hypothesise that tumorigenesis is also an evolutionary process, whereby the same Darwinian principles could be applied to elucidate the mechanisms responsible for cancer formation and development (Nowell, 1976). Owing to Nowell’s seminal work, a Darwinian framework has been historically adopted to develop models of tumour evolution and therapy resistance (Michor *et al*, 2004; Gatenby & Vincent, 2008; Pepper *et al*, 2009; Greaves & Maley, 2012) (see Box 1). While gene‐centric Darwinian principles have been shown to explain tumour evolutionary trajectories in multiple instances (Gerlinger & Swanton, 2010; Purushotham & Sullivan, 2010; Gillies *et al*, 2012), recent studies have suggested additional evolutionary concepts beyond Darwin’s are required to reconcile the full spectrum of evolutionary behaviours in cancer. Specifically, increasing evidence now supports macroevolutionary jumps as a feature of cancer (Stephens *et al*, 2011; Baca *et al*, 2013; Sottoriva *et al*, 2015), which are likely interspaced by phases of microevolutionary gradualism. Furthermore, evidence of discordant inheritance patterns between cells (Decarvalho *et al*, 2018), and the role of neutral evolution (Ling *et al*, 2015; Williams *et al*, 2016; Wu *et al*, 2016), cell plasticity (Pogrebniak & Curtis, 2018; Mills *et al*, 2019; Boumahdi & de Sauvage, 2020) and the tumour microenvironment (Coussens & Werb, 2002; Lin & Karin, 2007; Laconi *et al*, 2020) in cancer demand consideration of a broader set of evolutionary models. Understanding how tumour evolution influences disease progression and how such processes are shaped by environmental factors and treatment remains critical.

With this review, we discuss our understanding of tumour evolution as a process grounded in Darwinian selection but argue that in light of recent data, we must now incorporate these concepts into a larger conceptual framework inclusive of alternative approaches to fully understand, predict and better respond to cancer evolution and to improve patient outcome.

## Darwinian selection as the basis of subclonal diversity

Cancer has been historically viewed from a Darwinian gene‐centric perspective (Greaves & Maley, 2012). Indeed, tumours are frequently typified as a large population of genetically diverse cells giving rise to distinct subpopulations. Subclones will compete with one another for a limited set of nutrients and metabolites and face ever‐shifting selective pressures driven by both endogenous (*i.e*. microenvironmental pressures and geographical barriers) and exogenous (*i.e*. therapy) factors (Merlo *et al*, 2006). The outcome of this competition is the survival of clones adapted to grow under very specific conditions, as Darwinian selection is highly contextual and blind to the future. Many clones that were dominant at one point in time may reach evolutionary dead ends and disappear, while only a minority may be able to persist. Quoting Darwin “One general law, leading to the advancement of all organic beings, namely, multiply, vary, let the strongest live and the weakest die” (Darwin, 1859).

**Figure 1:**
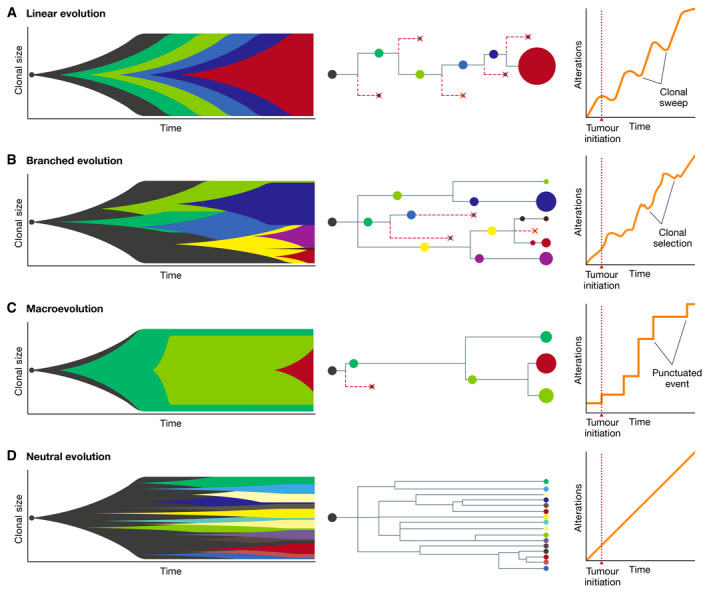
Models of tumour evolution Models of linear evolution (A), branched evolution (B), macroevolution (C) and neutral evolution (D) described by Muller plots representing dynamic changes in clonal size over time (left), clonal lineages and phylogenetic trees (centre) and changes in the number of alterations over time (right). Colours indicate different clones.

In the one to two decades, direct evidence to support Darwinian evolution in cancer has been reported, principally from studies using next‐generation sequencing (NGS) to perform detailed characterisation of genetic tumour evolution (see Box 2). One of the earliest studies was from Shah *et al* (2009), where matched primary and metastatic tissue from a lobular breast tumour were sequenced revealing extensive mutational heterogeneity with ∼80% of the non‐synonymous mutations in the metastasis absent from the primary site (Shah *et al*, 2009). The finding of pervasive heterogeneity in breast cancer has additionally been reported by the extensive work of Kornelia Polyak, which demonstrated that breast tumours were composed of a variety of cell types with distinct morphologies and behaviours, with the source of heterogeneity arising in part from clonal evolution (Campbell & Polyak, 2007). Early evidence of abundant, genetically diverse subpopulations of cells was also revealed through single‐cell sequencing (see Box 2) studies in breast cancer by Nick Navin and others (Navin *et al*, 2011). Regarding haematological malignancies, Anderson *et al*. were among the first to show branching evolutionary trajectories in acute lymphoblastic leukaemia (Anderson *et al*, 2011). Our own work from Gerlinger *et al* (2012) profiled 30 tumour samples from four renal cell carcinoma patients and revealed that 63 to 69% of all somatic mutations were not detectable across every tumour region (Gerlinger *et al*, 2012). These observations demonstrated the extent and relevance of branched evolution. Furthermore, evidence of parallel evolution was demonstrated for multiple tumour suppressor genes (*SETD2, PTEN, KDM5C*), suggesting selective pressures drive inactivation of the same gene multiple times within a single tumour. This report was followed by work from Nik‐Zainal *et al* (2012b), who studied the life history of 21 breast tumours, identifying extensive genetic variation within individual breast tumours (Nik‐Zainal *et al*, 2012b). This study also showed further evidence of selection, with each tumour containing a dominant subclonal lineage, representing more than 50% of tumour cells. Extending into further detail on the metastatic process, work from Gundem *et al* (2015) utilised autopsy sampling in 10 prostate cancer patients to identify metastasis to metastasis seeding as a common event (Gundem *et al*, 2015). This study emphasised not only the extent of subclonal diversification, but also the complexity of seeding routes to and between metastatic sites. However, these early studies were limited by small sample sizes. Furthermore, the diverse range of cancer types studied meant the nature of specific evolutionary patterns, as generalisable across all tumour types or histology specific, remained undetermined. Despite the limitations, these early NGS studies gave the first direct evidence of extensive genetic subclonal diversification, hence supporting a model of cancer growth as a branched evolutionary process (Fig 1). Furthermore, the demonstration of branched evolution as a feature of solid tumour growth spurred a change in thinking across the community to recognise the importance of Darwinian selection in cancer. Branched evolution has also been shown to be applicable to relatively homogeneous primary tumours and/or metastases, whereby particularly aggressive subclones that may achieve a clonal sweep and present clinically with a homogeneous profile (Reiter *et al*, 2018) (Fig 1). Clear examples of this are described in pancreatic cancer, where virtually all major driver gene alterations (*KRAS, CDKN2A, TP53, SMAD4*) are present in the most recent common ancestor and limited evidence of mutational heterogeneity is observed across metastases (Makohon‐Moore *et al*, 2017). Similar examples are observed in some aggressive renal cell carcinomas, where ∼10–20% of tumours exhibit multiple clonal driver mutations, limited heterogeneity and poor clinical outcome (Turajlic *et al*, 2018). It is proposed the variation in heterogeneity between tumours may reflect differences in the inherent biology of a given tumour and impact upon the process of metastatic dissemination and clinical outcome (Iacobuzio‐Donahue *et al*, 2020).

However, models of accumulating genetic changes subject to selective pressure are not fully sufficient to explain the full spectrum of cancer evolutionary histories, and increasing evidence points to the existence of non‐Darwinian mechanisms as important features of tumour evolution.

## Macroevolution and punctuated events

**Figure 2:**
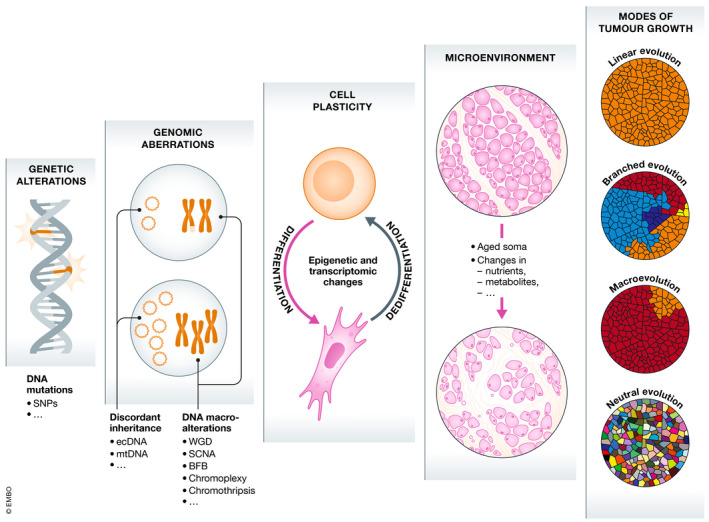
Scales of tumour evolution Schematic illustration of the different determinants of tumour evolution, which influence evolutionary trajectories through highly interdependent mechanisms, from a microscopic (left) to a macroscopic (right) scale.

Neo‐Darwinian models of tumour evolution generally assume that mutations are acquired sequentially in a gradual fashion over time. However, several lines of evidence suggest that in some cases, a large number of genomic aberrations may occur in short bursts of time in cancer cells (Stephens *et al*, 2011; Baca *et al*, 2013), as a consequence of chromosomal instability (CIN) (Bakhoum & Landau, 2017), breakage‐fusion‐bridge (BFB) cycles (Gisselsson *et al*, 2000), chromoplexy (Baca *et al*, 2013), chromothripsis (Stephens *et al*, 2011; Notta *et al*, 2016) and other similar catastrophic events (Fig 2). According to this model, tumour cells alternate long phases of relative mutational equilibrium with short periods of intense evolution, where tumour cells can acquire multiple strong driver events (Cross *et al*, 2016) (Fig 1). Such examples of saltatory evolution indicate that, at least in cancer, nature can under certain circumstances make jumps, contrary to what Darwin predicted. These observations are reminiscent of the “hopeful monsters” theorised by Richard Goldschmidt, *i.e*. organisms with a profound mutant genotype compared to their parents that hold the potential to establish a novel evolutionary lineage (Goldschmidt, 1941). Hence, through short and intense bursts of genomic change, cancer cells could potentially obtain greater fitness than would be possible through a gradual accumulation of alterations, owing to the simultaneous acquisition of multiple driver alterations (Korbel & Campbell, 2013). However, the phenotypic impact of such hereditary changes acquired through saltatory evolution, if any at all, will often be deleterious and only in rare instances will it result in an increase in cellular fitness and in the generation of viable “hopeful monsters” (Goldschmidt, 1941; Gerlinger *et al*, 2014b).

Regarding progression from primary tumour to metastasis and death, increasing evidence implicates macroevolutionary changes as important drivers of progression. For example, in the prospective TRACERx (TRAcking Cancer Evolution through therapy (Rx)) study (Jamal‐Hanjani *et al*, 2017), elevated copy number heterogeneity was identified as being most strongly associated with recurrence/death risk in non‐small cell lung cancer (NSCLC), whereas single nucleotide variant heterogeneity was non‐significant. Similarly, acquired aneuploidy was frequently detected in recurrent gliomas (Barthel *et al*, 2019), and genetic diversity alongside chromosomal complexity (characterised by high weighted genome integrity index (Endesfelder *et al*, 2014)) emerged as significant determinant of poor patient outcome in clear cell renal cell carcinoma (ccRCC) (Turajlic *et al*, 2018). In ccRCC, losses of chromosomes 9p21.3 (*CDKN2A*) and 14q31.1 (*HIF1A*) were specifically associated with reduced survival (Turajlic *et al*, 2018). The prognostic importance of macroevolutionary changes in the form of somatic copy number alterations (SCNAs), above point mutations, is now becoming increasingly recognised as a pan‐cancer phenomenon (Smith & Sheltzer, 2018). A major outstanding challenge however is minimal mapping of recurrent SCNA cytobands, to find specific causative genes. And even when clear driver genes emerge, as is likely the case for *CDKN2A* at 9p21 (Smith & Sheltzer, 2018), detailed functional work to delineate the precise mechanisms of disease progression remains to be completed. Additional evidence of punctuated evolution has been reported in prostate cancer, where intense genomic changes occurring over relatively few cataclysmic events, a process termed chromoplexy, were observed (Baca *et al*, 2013). Similarly, ER/PR/HER2 negative breast cancers were found to undergo short periods of evolution early in tumour development and to remain relatively stable at later stages (Gao *et al*, 2016). Tumour macroevolution was also found to be driven by chromothripsis, whereby a single catastrophic mutational event is thought to be responsible for the generation of highly complex genomic rearrangements involving dozens of breakpoints (Stephens *et al*, 2011). This process has been observed in several tumour types, such as bone cancers (Stephens *et al*, 2011), colon cancers (Kloosterman *et al*, 2011), neuroblastoma (Molenaar *et al*, 2012), glioblastoma (Malhotra *et al*, 2013) and pancreatic cancer (Notta *et al*, 2016). An extreme case of punctuated evolution, caused by the aforementioned mechanisms, is the “big bang” model, whereby a single or a limited number of catastrophic events and/or genomic crises occurring early in tumourigenesis are responsible for the generation of numerous intermixed subclones that will not substantially evolve over tumour progression due to weak selective pressure (Sottoriva *et al*, 2015). Such evolutionary dynamics were observed in several cancers, including colon cancers (Sottoriva *et al*, 2015) and hepatocellular carcinoma (Ling *et al*, 2015), as well as in pan‐cancer studies (Stephens *et al*, 2011).

Cancer evolution is conceptually similar to evolution in asexually reproducing organisms, whereby the impact of deleterious alterations in terms of fitness cannot be mitigated through sexual reproduction. A mechanism to alleviate the irreversible detrimental accumulation of alterations (*e.g*. extensive LOH events) may be whole genome doubling (WGD), a prevalent event in cancer (Storchova & Pellman, 2004; Zack *et al*, 2013; Dewhurst *et al*, 2014; Bielski *et al*, 2018) involving the doubling of the entire genome. The presence of additional, doubled wild‐type alleles as a consequence of WGD could allow the cell to better tolerate LOH events involving essential genes (López *et al*, 2020). The early occurrence of this event therefore creates a more tolerant and permissive environment which can fuel rapid genomic diversification and CIN, while at the same time may facilitate the sub functionalisation of duplicated genes (Storchova & Pellman, 2004; Huminiecki & Conant, 2012; Dewhurst *et al*, 2014). Consequently, WGD is often associated with high rates of chromosomal aberrations (Zack *et al*, 2013; Dewhurst *et al*, 2014) and with poor prognosis and intrinsic drug resistance (McGranahan *et al*, 2012; Bielski *et al*, 2018).

Importantly, certain classes of macroevolutionary events have been shown to be able to trigger other macroevolutionary events. For instance, chromothripsis is prone to arise in genomically unstable cells, such as those harbouring damaged telomeres or with hyperploidy (Mardin *et al*, 2015). Similarly, BFB cycles have been shown to generate increasing amounts of chromothripsis by providing free DNA ends that can engage in genomic rearrangement and by compromising centromere function (Umbreit *et al*, 2020). DNA replication stress has been shown to drive genomic instability by promoting both structural and numerical chromosomal aberrations (Burrell *et al*, 2013) and by triggering single nucleotide‐level mutagenesis mediated via APOBEC3B induction (Kanu *et al*, 2016), which in turn leads to incomplete replication of genomic DNA (Venkatesan *et al*, 2021). Relatedly, regional mutational clusters (kataegis) (Stephens *et al*, 2011) and lesion segregation (Aitken *et al*, 2020) have also been shown in some instances to be associated with chromothripsis and other rearrangement architectures (Nik‐Zainal *et al*, 2012a). The combination of such events rapidly accelerates tumour evolution, causing more non‐gradualism than any individual class by itself would.

## Discordant inheritance between cells

Recent studies have demonstrated that oncogene amplification within extrachromosomal DNA (ecDNA) is a frequent event in cancer (Verhaak *et al*, 2019). The existence of chromosomal material in cancer cells outside the autosomal genome has been long recognised, with the first reports of oncogenic ecDNAs going back as far as the 1980s, where sequences resembling *MYCN* were found in neuroblastoma cell lines (Kohl *et al*, 1983). However, it was only in the last few years that the frequency and functional relevance of ecDNAs in cancer started to be fully appreciated, thanks to the development of novel techniques such as long‐read whole‐genome sequencing and circular DNA library enrichment (Verhaak *et al*, 2019). ecDNAs are circular DNA structures located outside of chromosomes of variable size (ranging from 168 kb to 5 Mb, with a median size of 1.26 Mb) (Wu *et al*, 2019), which can contain one or more oncogenes (Bailey *et al*, 2020). ecDNAs provide cancer cells with a mechanism to achieve and maintain high copy oncogene amplification and diversity and to drive potent oncogene expression due to highly open chromatin, which allows for increased expression of genes encoded on circular DNA relative to their chromosomal counterparts (Wu *et al*, 2019; Kim *et al*, 2020). Importantly, the existence of ecDNAs defies Mendelian genetics. ecDNAs are replicated during S phase, but, owing to the lack of centromeres, they are subject to unequal segregation and are therefore randomly inherited by daughter cells during mitosis. As such, ecDNA‐based oncogene amplification can accelerate tumour evolution through non‐Mendelian mechanisms of inheritance and drive clonal expansion in genomically stable backgrounds (Decarvalho *et al*, 2018) (Fig 2). Importantly, the random distribution of ecDNAs fosters cell‐to‐cell variability in terms of both copy number and transcriptional levels of oncogenes, enabling tumours to acquire ITH more efficiently than through chromosomal amplifications (Turner *et al*, 2017; Verhaak *et al*, 2019). Several pan‐cancer studies revealed a high frequency of ecDNA (albeit at highly variable numbers) across tumour types, particularly in lung, breast and prostate cancer as well as glioblastoma and neuroblastoma (Fan *et al*, 2011; Turner *et al*, 2017; Deshpande *et al*, 2019; Bailey *et al*, 2020; Koche *et al*, 2020). Key oncogenes such as *MYC*, *MYCN*, *EGFR*, *PDGFRA*, *MET*, *HER2*, *DHFR*, *CDK4* and *MDM2* have been frequently observed on ecDNAs, suggesting that ecDNA‐mediated oncogene amplification is an important driver of tumour development and progression (Decarvalho *et al*, 2018; Gu *et al*, 2020). ecDNA amplification in cancer has been shown to increase cell proliferation, invasion and metastatisation and to negatively correlate with overall survival (Bailey *et al*, 2020). Consequently, ecDNA elimination has been shown to decrease oncogene amplification and to negatively affect cancer cell survival (Shimizu *et al*, 1998; Nathanson *et al*, 2014; Clarke *et al*, 2020; Oobatake & Shimizu, 2020). Furthermore, ecDNAs have been shown to enable tumour adaptation in response to changes in microenvironmental conditions and to selective pressure from therapy (Turner *et al*, 2017; Decarvalho *et al*, 2018; Kim *et al*, 2020), though in some instances ecDNA expression represents a cancer‐specific vulnerability (Nathanson *et al*, 2014).

## Neutral evolution

Neutral evolutionary models of cancer (Ling *et al*, 2015; Williams *et al*, 2016) are based on Motoo Kimura's work on population genetics which postulated that the vast majority of molecular alterations are not caused by selection but rather by random fixation of selectively neutral mutations through genetic drift (Kimura, 1983). According to the neutral tumour evolution model, cancer‐driving alterations are selected for and accumulate in a clonal fashion prior to tumour initiation, as a consequence of ageing and carcinogenic insults. Those alterations will be sufficient for tumour formation and development, with little to no contribution of alterations occurring over the course of cancer progression (Fig 1). Therefore, the genetic ITH observed in tumours may often be entirely the result of the random fixation of (nearly) neutral alterations in the population through genetic drift and as such have no functional role in promoting tumour growth and evolution. In one study, multi‐region sequencing of > 300 regions of a patient with hepatocellular carcinoma indicated that there was no particular selection for any clone within the tumour (Ling *et al*, 2015). In another study, subclonal allele frequencies from the TCGA cohorts were used to conclude that up to one‐third of all tumours do not show indications of subclonal selection but rather evolve in a neutral fashion (Williams *et al*, 2016). However, several lines of evidence suggest that these results may be an overestimation due to the low resolution of the data used for the study and may suffer from bias in the modelling, since variant abundance distributions may often not provide enough information to exclude selection (Tarabichi *et al*, 2018; Bozic *et al*, 2019).

Kimura's neutral theory essentially states that most alterations will be neutral, especially at low population sizes and with weak purifying selection. Most variants will not have any fitness effect, and the rare ones which will have an impact on fitness will predominantly be deleterious, as predicted by mathematical modelling (Cannataro *et al*, 2017). However, Kimura never excluded the importance of occasional strong positive selection in evolution, especially as a consequence of strong purifying selection (Kimura, 1983). Hence, when applying Kimura's neutral theory to cancer, selective pressures (*i.e.* microenvironmental changes, metastatisation, therapeutic intervention) must be taken into consideration. Therefore, while a treatment‐naïve tumour could at times evolve in a neutral fashion over the course of its progression, the emergence of strong selective forces, such as therapeutic pressure, may still drive the selection and expansion of previously neutral alterations (Almendro *et al*, 2014; Williams *et al*, 2016).

It is also worth noting that the presence of non‐cell‐autonomous drivers in small subpopulations may also give the false impression of neutral evolution (Marusyk *et al*, 2014). Indeed, Kornelia Polyak’s group demonstrated that tumours can at times be driven by a subclone that does not have higher fitness, but instead stimulates the growth of all tumour cells through non‐cell‐autonomous mechanisms (Marusyk *et al*, 2014). In such a scenario, it may be misleading to assume that the absence of a predominant clone in a tumour is evidence of neutral evolution, as non‐cell‐autonomous driving of tumour growth can maintain clonal diversity over clinically relevant time frames while simultaneously fuelling tumour growth and evolution.

## Non‐genetic determinants of evolution

There is increasing appreciation that tumour evolution is not only driven by mutations and genetic alterations but also from non‐genetic—often non‐heritable—determinants, such as cell plasticity and the tumour microenvironment (TME) (Caiado *et al*, 2016; Ramón y Cajal *et al*, 2020) (Fig 2).

### Cell plasticity

The notion that cancer cells can dynamically switch from one state to another in response to environmental stresses and therapeutic pressure without genomic alterations is gaining greater recognition (Pogrebniak & Curtis, 2018; Mills *et al*, 2019; Boumahdi & de Sauvage, 2020) (discussed in other reviews of this series by Milan *et al*, 2021). This phenomenon, termed cell plasticity, is characterised by a fundamental change in the biological properties of the cell occurring as a consequence of dynamic and reversible epigenetic and transcriptional changes (in sharp contrast to genetic alterations, which have binary and largely irreversible effects) (Calabrese *et al*, 2020). One of the main advantages of cell plasticity is the ability to swiftly react to dynamic changes in the tumour and in its microenvironment and to engage finely tuned and graded adaptive responses to stressors (*i.e*. inflammation and therapy) (Rambow *et al*, 2019). A classic example of cell plasticity is the epithelial–mesenchymal transition (EMT) (Nieto *et al*, 2016) (extensively covered by Brabletz *et al* (2021) in another review of this series). Cell plasticity allows for the rapid emergence of different cell states from the same genome, giving rise to a plethora of distinct phenotypes, a process that has shown to be promoted upon therapeutic intervention (Kemper *et al*, 2014; Gunnarsson *et al*, 2020; Marine *et al*, 2020). Cell plasticity has been extensively demonstrated as an adaptive mechanism to escape therapeutic pressure. One of the first evidence of such a mechanism was the identification of drug‐tolerant persisters (DTPs) cells emerging from drug‐sensitive NSCLC cell lines upon exposure to EGFR tyrosine kinase inhibitor (Sharma *et al*, 2010). This drug‐tolerant phenotype was shown to be transiently acquired and lost by individual cells, thereby demonstrating that cancer cells can dynamically and reversibly develop drug resistance through non‐genetic phenotype switch. Similarly, multiple phenotypically distinct—yet interdependent—drug‐tolerant populations were recently shown to emerge in melanoma PDX models in response to MAPKi treatment through adaptive non‐genetic mechanisms (Rambow *et al*, 2018). Importantly, although resistant phenotypes are often non‐heritable, they can protect the tumour cell population from eradication thereby increasing overall tumour fitness through non‐Darwinian mechanisms (Kemper *et al*, 2014; Gunnarsson *et al*, 2020; Marine *et al*, 2020). Cell plasticity can therefore enable the emergence of more permanent resistance mechanisms, as was shown in melanoma, whereby therapeutic pressure initially caused the emergence of a transient drug‐tolerant transcriptional state which was later converted into a stably resistant phenotype (Shaffer *et al*, 2017).

### Tumour microenvironment

The observation that healthy tissues often display pervasive somatic mutations, even in cancer driver genes, suggests that genetic mechanisms alone may be not be sufficient to drive malignant transformation (Martincorena *et al*, 2015, 2018; Teixeira *et al*, 2019; Yizhak *et al*, 2019; Yoshida *et al*, 2020). It has been long noted that the incidence of most cancers uniformly increases after 50 years of age (Siegel *et al*, 2018b), regardless of the many differences in terms of their cellular origin, of the number of alterations required for their initiation and of the external risk factors (such as smoking, alcohol and UV light) (Rozhok & De Gregori, 2019; Laconi *et al*, 2020). To explain this discrepancy, James DeGregori’s group demonstrated that cancer incidence rapidly increases with age not only as a consequence of the accumulation of alterations but also (if not predominantly) because of the age‐dependent physiological decline of the soma, combined with a general weakening of the immune system which is less efficient at eliminating altered (precancerous) cells (DeGregori *et al*, 2020; Laconi *et al*, 2020). While a young healthy tissue environment may continuously and effectively prevent tumorigenesis, an aged one would provide fertile ground for cancer formation instead. Permissive environments have been shown to result in population expansion in nature, whereby relaxed constraints are known to often spur the establishment, expansion and persistence of particularly versatile species. For instance, a permissive environment was shown to have led to the expansion of two species of parasites in the Arctic as a consequence of climate change (Kutz *et al*, 2013; Kafle *et al*, 2020).

Intriguingly, the effect of mutagenic agents was also found to be influenced by the tissue type, as the mutational burden varied greatly based on tissue, suggesting tissue‐specific differences in toxicokinetic, DNA repair activity or of the TME (Riva *et al*, 2020). Similar evidence comes from the observation that genetic diseases driven by highly penetrant mutations predisposing to cancer invariably lead to very specific cancer types, although the same mutation is shared between all the cells of the organism. This is the case of the *NF1* mutation in neurofibromatosis, which often leads to tumours of the nervous system (Gutmann *et al*, 2017), of the *TP53* mutations in the Li‐Fraumeni syndrome, which predispose to breast cancer, adrenocortical carcinomas, central nervous system tumours, osteosarcomas and soft‐tissue sarcomas (Malkin, 2011), and of the *BRCA1/2* mutations, which considerably increase the risk of breast cancer (Tung & Garber, 2018). Hence, even highly penetrant cancer‐driving alterations may not be sufficient to drive cancer formation per se. Instead, these examples suggest that oncogenic mutations may often confer a cell type‐specific advantage in terms of fitness rather than a generic selective advantage on all cells and that very specific environmental conditions are needed for tumour initiation. The observation that certain cancer types or cancer subtypes metastasise to specific locations, a fact that cannot be solely be explained by circulation patterns, further reinforces the idea that tumours require particular environments for their growth and survival, even in later stages of cancer evolution (Nguyen *et al*, 2009). These are only but a few examples of how the TME can affect cancer initiation and evolution, as this topic is amply discussed in other reviews of this series (see Kepp *et al*, 2021; Parker *et al*, 2021).

## Clinical opportunities: leveraging evolutionary principles

Advances over the last decade have led to a deeper understanding of the molecular pathogenesis of cancer and of its evolution. Small‐molecule inhibitors, such as BRAF and EGFR inhibitors (Bedard *et al*, 2020), and immune checkpoint inhibitors (CPI), such as anti‐PD‐1/L1 and anti‐CTLA‐4 (Waldman *et al*, 2020), have revolutionised cancer therapy (Chen *et al*, 2019). However, despite the significant benefits brought about by these approaches, many advanced tumours—even those with marked initial responses—rapidly develop resistance (Gatenby & Brown, 2020). The dynamic nature of tumour evolution, especially under the strong selective pressure exerted by anti‐cancer therapies, makes longitudinal monitoring necessary (see Box 3) and warrants flexible therapeutic strategies based on the evolutionary changes within a patient's tumour.

### Limiting tumour adaptation

**Figure 3:**
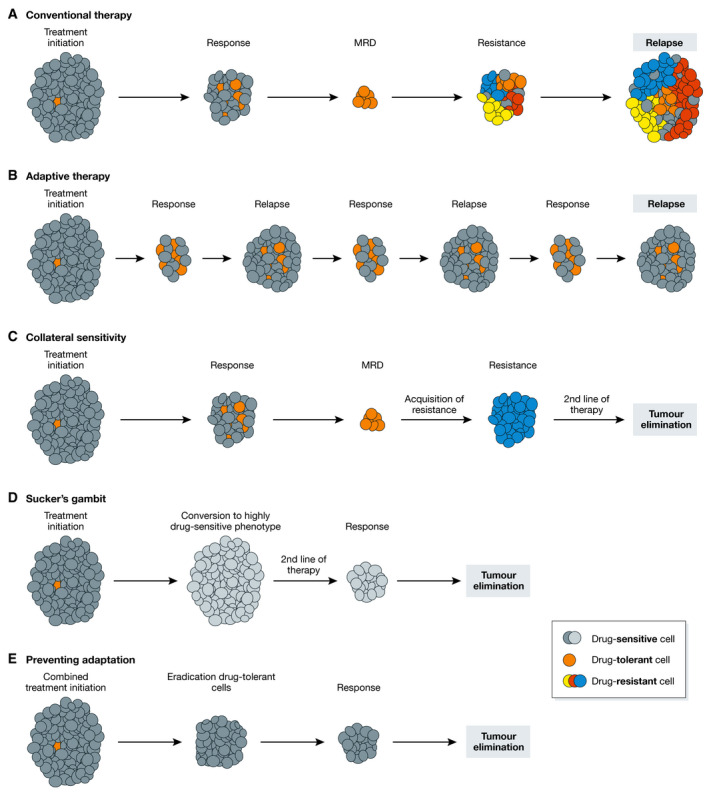
Evolutionary‐based treatment strategies (A) Most conventional therapeutic approaches will lead to marked initial responses but often fail to eliminate—or induce the emergence of—drug‐tolerant cells. This population of minimal residual disease (MRD) represents a reservoir of malignant cells from which drug‐resistant clone(s) may emerge and cause tumour relapse. (B) In adaptive therapy, treatment is discontinued upon reaching a pre‐defined threshold so as not to eliminate all drug‐sensitive cells (dark grey). These cells are then allowed to grow back and are expected to suppress the growth of drug‐tolerant (or therapy‐resistant) clones owing to their higher fitness upon withdrawal. (C) Collateral sensitivities, wherein acquisition of resistance to a first drug induces susceptibility to a second one, can be exploited to target cancer‐specific vulnerabilities and potentially lead to tumour eradication. (D) In the Sucker’s gambit, the first drug forces a phenotypic adaptation that pushes tumour cells into an evolutionary trap by increasing the fitness of a highly drug‐sensitive population (leading to its clonal dominance), which is then eliminated by a second line of treatment. (E) Limiting drug adaptation may prevent the establishment of drug‐tolerant (MRD) and drug‐resistant populations. In this setting, a combination of drugs is used to simultaneously eradicate tumour cells and to prevent the emergence of drug‐induced adaptive responses.

Increasing evidence indicates that both Darwinian and non‐Darwinian adaptive mechanisms can be activated shortly after therapy initiation to cope with the stress caused by the treatment (Hata *et al*, 2016; Rambow *et al*, 2018; Russo *et al*, 2019; Vendramin *et al*, 2021). The employment of such responses thereby fosters the emergence of a reservoir of drug‐tolerant cellular populations from which therapy‐resistant clones can evolve from. Therefore, therapeutic strategies targeting the mechanisms responsible for drug tolerance may prevent, or at least delay, the evolution of acquired resistance (Fig 3). This strategy was tested in a recent study where the selective pressure exerted by EGFR inhibitors in lung cancer was shown to induce APOBEC3B overexpression, leading to increased cancer cell mutability, fostering tumour adaptation to treatment (preprint: Mayekar *et al*, 2020). Similar findings were reported in colon cancer, whereby drug‐tolerant cells were shown to temporarily down‐regulate mismatch repair (MMR) and homologous recombination DNA repair genes in favour of error‐prone polymerases as an adaptive response to overcome BRAF and EGFR inhibition (Russo *et al*, 2019). These findings suggest that, similar to bacteria (Luria & Delbrück, 1943), cancer cells can temporarily enhance their mutability and genetic instability as an adaptive response to overcome therapeutic pressure. Inhibition of these adaptive mechanisms could therefore abolish drug‐driven adaptive mutagenesis and potentially prevent the emergence of drug‐resistant populations.

Additionally, there is growing appreciation that non‐Darwinian mechanisms can drive tumour adaptation, especially in the context of therapy resistance, where a clear genetic cause for therapy failure has only been identified in about 60% of tumours. For instance, MAPK inhibition was shown to drive transcriptional reprogramming in melanomas shortly after treatment initiation which resulted in drug tolerance (Smith *et al*, 2016). Importantly, this response was reversible and non‐mutational in nature. Targeting of a master regulator of this adaptive mechanism was shown to prevent drug tolerance and re‐sensitise melanoma cells to MAPKi (Smith *et al*, 2016). Similarly, the activation of the integrated stress response (ISR) was observed to foster drug tolerance in melanoma. In this context, ISR activation was found to indirectly enhance mitochondrial translation, making these cells highly vulnerable to mitochondrial translation inhibitors, which were shown to reduce tumour heterogeneity and to prevent acquisition of resistance regardless of the tumour mutational profile or phenotype (Vendramin *et al*, 2021). The TME was also shown to promote tumour drug tolerance. For instance, paradoxical activation of the fibroblastic stroma in BRAF inhibitor treated melanomas resulted in enhanced matrix remodelling. The remodelled matrix was found to promote BRAF‐independent ERK activation through FAK signalling, thereby enabling drug tolerance and melanoma cell survival. Concomitant inhibition of BRAF and FAK prevented ERK reactivation and led to tumour volume stabilisation (Hirata *et al*, 2015). Similarly, inhibition of the microenvironment‐produced morphogen EDN3 abrogated transcriptional reprogramming and phenotype switch in melanomas and reduced tumour aggressiveness and growth (Kim *et al*, 2017).

### Exploiting collateral sensitivities

The paradox of treating dynamically evolving cancers is that therapeutic strategies are typically administered in a fixed, sequential schedule in the hope of reducing tumour burden (Gatenby *et al*, 2009). The integration of evolutionary principles in the design of anti‐cancer therapies may therefore bring about substantial clinical benefits. Knowledge of tumour evolutionary responses to therapy could allow the anticipation of relapse by pre‐emptively targeting drug resistance mechanisms or to exploit collateral sensitivities, *i.e*. specific weaknesses caused by the development of resistance to a particular treatment (Pluchino *et al*, 2012; Efferth *et al*, 2020) (Fig 3). For instance, combining different classes of ABL1 inhibitors with mutually exclusive profiles of resistance mutations in chronic myeloid leukaemia can prevent the emergence of drug‐resistant subclones and lead to durable responses *in vivo* (Zhao *et al*, 2016; Wylie *et al*, 2017). Another example is gefitinib‐resistant NSCLC, which shows collateral sensitivity for TNFα when compared to the non‐resistant counterpart (Ando *et al*, 2005). A similar strategy is the “sucker's gambit”, in which the initial therapy, rather than killing all cancer cells, aims to push them into an evolutionary *cul‐de‐sac* by forcing a phenotypic adaptation that is then exploited in second‐line therapy (Merlo *et al*, 2006; Schweizer *et al*, 2015) (Fig 3). The first drug will affect most subpopulations and promote the emergence and clonal dominance of a specific clone, which would then be eliminated by the addition of a second drug (West *et al*, 2019). A limitation of this approach is that it is based on the premise that all cells will develop the same mechanism of resistance, whereas a plethora of *in vivo* experiments and autopsy studies have demonstrated that most individual tumours can achieve resistance (to chemo‐, targeted and immunotherapy) via multiple distinct routes simultaneously, a process termed polyclonal resistance (Burrell & Swanton, 2014; Shi *et al*, 2014; Kemper *et al*, 2015; Faltas *et al*, 2016; Ascierto *et al*, 2017; Rambow *et al*, 2018; Razavi *et al*, 2018; Krook *et al*, 2019a; Rosenthal *et al*, 2019; Sanchez‐Vega *et al*, 2019).

An alternative strategy, named “benign cell booster”, aims instead to promote the growth of benign, less aggressive tumour subpopulations at the expense of highly malignant populations (Maley *et al*, 2004). Importantly, such an approach would prevent the emergence of aggressive treatment‐resistant subpopulations and have likely limited adverse effects since it would not be aimed at killing cancer cells but rather select for particularly benign tumour cell populations.

### Adaptive therapies

Another intriguing therapeutic approach based on evolutionary principles is adaptive therapy, a therapeutic strategy with the goal of maintaining a stable tumour population below a certain symptomatic threshold, rather than trying to achieve complete tumour elimination, through the use of repeated ON/OFF treatment cycles (Gatenby *et al*, 2009) (Fig 3). This strategy aims to make use of the understanding of the mechanisms of therapy resistance as well as their “cost” and to exploit the differences in terms of fitness between drug‐sensitive and drug‐resistant subpopulations (Cunningham, 2019). In such a scenario, the optimal dose and treatment duration is not the maximum possible but rather the minimum necessary. In essence, a treatment cycle would last until tumour response (*i.e*. regression below a certain, pre‐defined threshold) is observed, after which therapy is discontinued. This approach enables minimal tumour cell proliferation to prevent the elimination of all treatment‐sensitive tumour clones, which are expected to have a competitive advantage in terms of fitness over treatment‐resistant populations. Upon relapse, the tumour should mostly be comprised of treatment‐sensitive cells; therefore, a new treatment cycle will be initiated to bring the tumour volume below a symptomatic threshold. Despite its initial promises and successes in *in vitro* and *in vivo* models (Enriquez‐Navas *et al*, 2016; Bacevic *et al*, 2017), this strategy has not achieve notable results in the clinic, as indefinite tumour control, the goal of such an approach, has not been achieved (Crook *et al*, 2012; Hussain *et al*, 2013; Algazi *et al*, 2020). One limitation of this approach is the lack of tools enabling constant monitoring of tumour subclonal evolution (*e.g*. via ctDNA/CTCs, see Box 3) over the course of treatment. Another weakness of this strategy is the assumption that drug resistance always comes at a fitness “cost” for the cell, which may not be true in all cases, especially in the context of polyclonal resistance, where different resistance mechanisms may differentially impact cell fitness. Indeed, there is evidence that acquisition of resistance, at least in some instances, may not be associated with a fitness cost at all (Shah *et al*, 2007; ffrench‐Constant & Bass, 2017; Strobl *et al*, 2021).

### Early detection

Many early‐stage cancers can be cured by surgery alone if removed before metastasis has occurred, by surgery ± adjuvant therapy. Past that point, these approaches are seldomly curative (Siegel *et al*, 2017; Cohen *et al*, 2018). Therefore, until more potent and specific therapeutic agents are developed, a promising strategy for reducing cancer mortality is early tumour detection, which has been shown to reduce cancer mortality in several cancer types including lung, breast, bladder, colorectal, head and neck and testicular cancer as well as melanoma (Hawkes, 2019; Lee *et al*, 2019; Pantel & Alix‐Panabières, 2019; Crosby *et al*, 2020; de Koning *et al*, 2020). Early‐stage cancer detection may also allow the identification of tumours in early evolutionary stages, when they may be less resilient to treatment due to their more homogeneous nature (Jamal‐Hanjani *et al*, 2015). Additionally, detecting pre‐existing therapy‐resistant clones before treatment initiation may allow the employment of individualised therapeutic approaches aimed at eliminating drug‐tolerant/resistant populations. A major evolutionary question, that in the context of early detection remains unaddressed, is timing of tumour initiation and the lead‐time between malignant transformation and clinical presentation. Evolutionary tools such as the molecular clock, originally introduced by Emile Zuckerkandl and Linus Pauling in the 1960s (Morgan, 1998), have recently provided some preliminary estimates using cancer genomics datasets. These approaches take advantage of the somatic mutations acquired by cancer cells with clocklike properties, *i.e*. a correlation with the chronological age of the person. With this knowledge, mutation counts can be used to infer the timing of biological events, for example 3p loss/5q gain events found in 36% of ccRCC patients were timed back several decades to childhood/adolescence (Mitchell *et al*, 2018). In another study, a primary colorectal tumour was timed to have likely emerged 5 to 8 years before clinical diagnosis (Lote *et al*, 2017). In another case, analysis of early NSCLC revealed WGD events and truncal driver events in ex‐smokers dating back more than 20 years prior to tumour detection, indicating a prolonged tumour latency period from the first oncogenic insults to clinical manifestation (De Bruin *et al*, 2014). Recent large‐scale work from the Pan‐Cancer Analysis of Whole Genomes (PCAWG) consortium in 2,658 tumours also revealed that driver mutations often precede clinical diagnosis by many years, if not decades (Gerstung *et al*, 2020). While inferred in nature, these studies still provide accumulating evidence that tumour initiation likely dates back years or even decades before clinical presentation, hence suggesting an extended window for cancer early detection, likely through the use of non‐invasive blood‐based tests (see Box 3) (Liu *et al*, 2020). In addition to genomic data, information on the history of prior mutagenic exposures (*i.e*. duration of smoking, age of smoking cessation) can be additionally utilised to validate and refine timing inferences (De Bruin *et al*, 2014). Lastly, as well as absolute timing, the accumulation of mutations can also be timed relative (*i.e*. before or after) macroevolutionary events such as WGD (López *et al*, 2020).

### Minimal residual disease

Particular attention should also be given to minimal residual disease (MRD) detection and characterisation. MRD represents latent disease*, i.e*. a small population of undetectable malignant cells that persist or recur following initial surgical or systemic treatment. These cells may represent a reservoir of metastasis competent or drug‐tolerant cells able to drive future tumour relapse (Luskin *et al*, 2018; Rambow *et al*, 2018; Chin *et al*, 2019). Owing to the limited number of cells, it is technically challenging to detect MRD through conventional approaches, such as computed tomography scans. To date, the most promising method to detect MRD is achieved through circulating tumour DNA assessment (see Box 3). This approach has been shown to be successful in several studies in bladder cancer (Dudley *et al*, 2019), NSCLC (Abbosh *et al*, 2017; Chaudhuri *et al*, 2017), colon cancer (Tie *et al*, 2016), breast cancer (Mounier *et al*, 2015), oesophageal cancer (Azad *et al*, 2020) and a range of other solid tumour types (Chin *et al*, 2019). Importantly, macroevolutionary changes caused by large chromosomal aberrations, WGD or CNAs that appear during metastatic formation have been detected through analysis of circulating tumour cells (CTCs) and circulating tumour DNA (ctDNA) in recent studies performed on colon cancer patients with MRD (Gao *et al*, 2017; Joosse *et al*, 2018). Additionally, post‐surgical detection of MRD is a strong predictor of impending disease relapse (Abbosh *et al*, 2018; Luskin *et al*, 2018; Azad *et al*, 2020). Hence, rational targeting of MRD is emerging as a promising strategy to prevent therapy resistance and relapse (Luskin *et al*, 2018). There are several advantages to treating patients with MRD rather than waiting for clinical relapse to initiate further therapy. First, tracking of MRD allows the identification of alterations emerging in response to treatment. Second, earlier detection of relapse via MRD allows further treatment to be implemented earlier, where a window of more favourable outcome may be achievable when tumour burden and ITH will be lower. Accordingly, the design of personalised ctDNA panels based on patient mutational status could provide a patient‐specific biomarker of tumour response to treatment. For instance, a recent study demonstrated that the use of a personalised ctDNA panel led to detection of breast cancer metastatic recurrence up to two years prior to overt clinical manifestation (Coombes *et al*, 2019). Last, this approach could allow to adapt therapeutic regimens in the adjuvant setting to eliminate all persisting cells. Regarding predictive MRD tools, the PROSPECT‐C study demonstrated that time to anti‐EGFR treatment failure could be reliably predicted using longitudinal ctDNA sampling in colorectal cancer, hence generating a window of opportunity for future intervention (Khan *et al*, 2018). Similarly, the IMvigor010 study (Powles *et al*, 2021) demonstrated that MRD detection through ctDNA detection, using the Signatera assay co‐developed by our laboratory and Natera (Abbosh *et al*, 2017), could be used to stratify patients who will benefit from atezolizumab (anti‐PD‐L1) postoperatively. Evidence that targeting MRD can prevent relapse comes from the experience of treating patients with completely resected NSCLC (Winton *et al*, 2005), colon cancer (André *et al*, 2009) and breast cancer (Albain *et al*, 2012) with adjuvant (or neoadjuvant) therapy, with promising results coming from *in vivo* experiments in melanoma (Rambow *et al*, 2018; Marin‐Bejar *et al*, 2021; Vendramin *et al*, 2021). A similar approach is currently being tested in the MERMAID‐1 Phase III clinical trial where the effects of adjuvant treatment with durvalumab (anti‐PD‐L1) plus chemotherapy versus chemotherapy alone on disease‐free survival (DFS) in patients with completely resected stage II‐III NSCLC who show evidence of MRD.

## Conclusion and future perspectives

The longstanding assumption that cancer evolution can only be viewed through a Darwinian lens has been shown to be insufficient to explain the complex evolutionary patterns observed in human malignancies. While our understanding of tumour evolution has been built on the foundations of Darwinian selection, increasing evidence indicates that cancer evolutionary dynamics often defy Darwinian principles. The observation that tumours can evolve in a punctuated fashion, rather than in a gradual fashion as Darwin theorised when he stated that “natural selection can act only by taking advantage of slight successive variations; she can never take a leap, but must advance by the shortest and slowest step” (Darwin, 1859), demonstrates that nature does indeed make macroevolutionary jumps. It is also important to consider that the diversity within tumours is not necessarily selectively driven but can also emerge in some tumours as the result of neutral evolutionary dynamics. Evidence of discordant inheritance patterns between cells represents additional proof of the relevance of non‐classical mechanisms in cancer evolution. Furthermore, neo‐Darwinian evolutionary models are traditionally based on a gene‐centric view of evolution, whereas increasing evidence highlights the role of non‐genetic contributors, such as cell plasticity and the tumour microenvironment, as key determinants of cancer evolution. Last, it is becoming increasingly clear that tumorigenesis may in some cases reflect the physiological decline of a tissue, whereby an ageing soma provides a more permissive environment for malignant transformation. It is important to note that individual tumours may not follow a single evolutionary model. Multiple mechanisms may instead be operating either concomitantly or at different stages of progression. Hence, a coherent and unified approach encompassing Darwinian and non‐Darwinian evolutionary theories is needed to fully comprehend the breadth and the depth of cancer evolutionary dynamics.

Research output thus far has revealed that tumour evolution is highly varied in its nature, as well as evidenced how little we still know about cancer evolutionary trajectories. While the studies discussed above have provided critical clues on the driving forces of tumour evolution, they were generally based on individual time points and limited in terms of resolution and cohort size. Larger clinical studies, harnessing the full potential of recent technological innovations, such as single‐cell approaches, making use of multiple longitudinal and post‐mortem approaches, such as blood‐based tests monitoring ctDNA/CTCs and rapid research autopsies, respectively, are thus warranted. The implementation of these approaches will allow us to shed light on the dynamics of cancer evolution and to develop evolutionary‐guided therapies to impact clinical outcomes.

In conclusion, understanding cancer requires an evolutionary perspective. The study of malignant transformation within an evolutionary framework has potential to bring about a substantial improvement in clinical outcomes, by providing novel insights to support personalised cancer treatment, as well anticipating and proactively managing therapeutic resistance.

Box 1 **An historical perspective of tumour evolution and intratumour heterogeneity**

One hundred years have passed since Theodor Boveri set the foundations for much of our understanding of the origins of cancer. He not only hypothesised that cancers arise from normal cells as a consequence of genetic alterations, but also postulated that most tumours and their metastases originate from one cell, a concept that shaped today’s understanding of clonal expansion (Boveri, 2008). The application of evolutionary concepts to understand cancer formation and development can be attributed to Peter Nowell, who pioneered the hypothesis of tumour evolution. Nowell’s model stated that most cancers originate from a single neoplastic cell and evolve through a process of selection for somatic alterations, leading to the proliferation and survival of the most aggressive clones (Nowell, 1976). Despite this conceptual advance, Nowell’s view was largely overlooked, and tumour evolution was traditionally viewed as a linear succession of clonal cell divisions. Under the linear model, alterations accrue in progenitor cells in a stepwise fashion and endow cells with a strong selective advantage, hence enabling previous clones to be outcompeted (Fig 1). As a result, tumours would be composed of clonally identical cells resulting from continuous selective sweeps (Davis *et al*, 2017). Gloria Heppner challenged this view by demonstrating that tumours are comprised of genetically different subclones exhibiting fundamentally distinct behaviours (Dexter *et al*, 1978). By applying concepts of population genetics, she described tumours as “societies highly adapted for survival” and recognised that tumours “survive natural and artificial (therapeutic) selection through heterogeneity by producing new variants to "outflank" it.” (Heppner, 1984). These observations supported a model whereby tumours grow in a non‐linear, branched fashion, with multiple subclones derived from a common ancestor eventually diverging and expanding simultaneously with differing fitness (Greaves & Maley, 2012; Swanton, 2012) (Fig 1).

A consequence of branched tumour evolution is intratumour heterogeneity (ITH)—that is the coexistence of molecularly and phenotypically distinct subclones within a tumour. Tumour morphological heterogeneity has been long recognised by pathologists such as Johannes Muller and Rudolf Virchow in their pioneering studies in the 19^th^ century as well as Theodor Boveri (Muller, 1973; Boveri, 2008; Parquet, 2014; Wright, 2014). Those initial discoveries were substantiated by several groups with orthogonal techniques, which provided evidence for intratumour diversity across multiple cancer types (Shapiro *et al*, 1981; Teixeira *et al*, 1996; Takahashi *et al*, 1998; Maley *et al*, 2006; Cottu *et al*, 2008). However, it was only with the recent technological innovations (see Box 2), particularly next‐generation sequencing (NGS), that the full breath of ITH could start to be fully resolved (Campbell *et al*, 2010; Navin *et al*, 2010; Gerlinger *et al*, 2012; Nik‐Zainal *et al*, 2012b; Walter *et al*, 2012; Patel *et al*, 2014; Sottoriva *et al*, 2015; Jamal‐Hanjani *et al*, 2017; Naxerova *et al*, 2017; Turajlic *et al*, 2018; Fittall & Van Loo, 2019; Rambow *et al*, 2019; Reiter *et al*, 2019; Gerstung *et al*, 2020; Machnik & Oleksiewicz, 2020; Marine *et al*, 2020).

Box 2 **Sequencing technologies to assess tumour evolution**

The advent of next‐generation sequencing (NGS) has revolutionised biology. Its high‐throughput, scalability, speed, and cost efficiency enabled researchers to study the genomic and transcriptomic profiles of many human diseases, including cancer, with unprecedented resolution. Studies of cancer evolutionary dynamics and ITH have particularly benefited from these technological advances.

**Bulk** **sequencing**

To date, most studies of tumour evolutionary dynamics have relied on the sequencing of bulk tumour samples. While this methodology can only provide an indirect measurement of the subclonal composition of a tumour biopsy, tumour phylogenetic architecture can still be inferred through computational approaches. By sequencing samples at sufficiently high coverage depth, variant allele frequencies (VAFs) can be quantified. VAFs can then be further corrected for tumour purity and local copy number state to derive cancer cell fraction (CCF) values, which intuitively measure what proportion of cancer cells bear a given mutation. CCFs can then be clustered using a Dirichlet process to identify clonal and subclonal populations—based on the assumption that mutations with similar CCFs fit together to form genetically distinct populations. Once the subclonal architecture of a given tumour is defined (*i.e*. the complete set of detected clones/subclones), phylogenetic techniques can be used to infer the most likely parent to child ordering relationships. This enables phylogenetic tree construction, with each tumour (sub)clone being placed on its relevant branch. Phylogenetic tree construction must adhere to basic principles, such as the pigeonhole principle, which stipulates that if there are *m* containers (pigeonholes) and *n* items (pigeons) to go within the containers and if *n* > *m,* then there must be a container with more than one item. This principle ensures that the CCF sum of child subclones cannot exceed that of its parental ancestor. Computationally, these methods are implemented within a wide number of tools, and consensus efforts are seeking to drive greater standardisation in methodologies (Schwartz & Schäffer, 2017). It should be noted that key information needed for reconstructing tumour evolution may however be lost in the process, due to confounding effects derived from dealing with mixed cell populations. For instance, alterations only present in low frequency small subclones will likely be confused as noise, posing a significant limitation for tree reconstitution (Cibulskis *et al*, 2013). Additionally, deconvolution of bulk sequencing data is needed to infer evolutionary dynamics, an approach that may limit the identification of branching events (Malikic *et al*, 2019).

**Single‐cell** **approaches**

The recent development of single‐cell approaches (SCA) has allowed the analysis of the genomes, transcriptomes, epigenomes, proteomes and even metabolomes of individual cancer cells, as well as the TME, with unprecedented resolution (Navin *et al*, 2011; Hiley *et al*, 2014; Wang *et al*, 2014; Navin, 2015). The most widely used SCA has been single‐cell RNA sequencing (scRNA‐seq), which has recently emerged as a valuable tool to study tumour evolutionary dynamics (Nam *et al*, 2021). Owing to its high resolution, scRNA‐seq permits detection of genes expressed even in small subclones which would be missed by bulk RNA‐seq, allowing, for instance, the identification of minor treatment‐resistant cell populations contributing to therapy failure (Rambow *et al*, 2018). Additionally, copy number variation and loss of heterozygosity in individual cells can sometimes be inferred through scRNA‐seq, although in most cases the resolution is not sufficient for novel SCNA discovery (Fan *et al*, 2018). Moreover, by obtaining transcriptomic data from hundreds of individual cells in a range of different evolutionary stages, cells can be ordered in pseudotime and within evolutionary trajectories. This allows the inference of tumour evolutionary history and dynamics, albeit without providing information regarding the direction of such trajectories. To overcome this limitation, RNA velocity analyses can be used. By measuring the relative ratio between intronic and exonic reads, the rate of change in transcript abundance can be inferred, thus providing an estimate of the future transcriptional state of a cell alongside with a better understanding of the cellular transcriptional dynamics (La Manno *et al*, 2018). Another SCA approach that has emerged in the last decade is single‐cell DNA sequencing (scDNA‐seq), which can provide genomic profiles of individual cells, thereby allowing the inference of genetic phylogeny without any form of deconvolution. Despite its promises, scDNA‐seq still has major limitations. First, the coverage obtained with such approaches tends to be low because of the limited amount of DNA in a single cell, which typically contains a few picograms of genomic DNA, whereas NGS requires nanogram amounts of starting DNA for library preparation. Therefore, a critical step for single‐cell sequencing is whole‐genome amplification to generate sufficient DNA for library construction, a process which is rather error‐prone (Chen *et al*, 2018). Other frequent issues are an increase rate of false negatives due to allelic dropout and additional noise stemming from accidentally sequencing doublets (Pugh *et al*, 2008; Lasken, 2013; Navin, 2014; Van Loo & Voet, 2014; Simonsen *et al*, 2018; Mallory *et al*, 2020). Interestingly, the weaknesses of scDNA‐seq can be complemented by bulk DNA sequencing and vice versa and the use of both data types for inferring phylogeny of tumours has been shown to provide more accurate results and increased resolution in capturing ITH complexity and evolutionary dynamics (Salehi *et al*, 2017; Malikic *et al*, 2019).

Box 3 **Sampling techniques to study tumour evolution**

**Multiregional (longitudinal) biopsies**

Over the last few years, increasing efforts have been put towards sequencing multiple geographically distinct areas of the same tumour and of its metastases and/or samples taken from the same patient at different time points (McGranahan & Swanton, 2015; Chkhaidze *et al*, 2019). These analyses have proven to be critical for the reconstruction of tumour evolutionary histories, revealing extraordinarily complex and dynamic phylogenetic architectures of cancer subclones and their variegated genetic, epigenetic and transcriptomics profiles. However, multiregional and longitudinal sampling through invasive biopsies is often deemed clinically unfeasible, since the performance status of the patient and the location of the tumour frequently prevent such an approach (Turajlic *et al*, 2018; Chkhaidze *et al*, 2019; Litchfield *et al*, 2020).

**Rapid** **research autopsy**

Rapid research autopsy is gaining interest as a powerful technique to assess ITH and tumour evolution through improved tissue sampling, with the potential to overcome the limitations of sampling in living patients in several ways (Duregon *et al*, 2019). First, they allow the collection of large quantities of tumour tissue, as well as the TME, from multiple body sites from individual patients, supporting a more complete representation of metastatic lesions. Second, enough material can be collected to be used for multiple orthogonal studies (Avigdor *et al*, 2017; Pisapia *et al*, 2017; Duregon *et al*, 2019). Third, these samples can be used for the development of patient‐derived xenografts, organoids and primary cell lines, thus allowing mechanistical validation of the data generated through orthogonal analyses (Grasso *et al*, 2012; Xie *et al*, 2015; Krook *et al*, 2019b). Last, the samples obtained are typically composed of therapy‐resistant cells and can inform about the evolution of drug resistance (Dagogo‐Jack & Shaw, 2018). Such an approach offers an ethical alternative to multiregional biopsy sampling of living patients and can be effectively used to overcome its limitations. Albeit limited in number, the studies performed through rapid research autopsies have substantially increased our understanding of ITH and tumour evolution, and how both processes are affected by therapeutic selective pressure (Campbell *et al*, 2010; Haffner *et al*, 2015; Hoadley *et al*, 2016; Brown *et al*, 2017; Siegel *et al*, 2018a; De Mattos‐Arruda *et al*, 2019). For instance, rapid research autopsies have allowed tracing of the origins of metastases to individual subclones, delineating a precise time course from an initial founder cell to the development of metastases (Yachida *et al*, 2010; Gerlinger *et al*, 2012; Aryee *et al*, 2013; Gerlinger *et al*, 2014a). It is worth noting that, apart from some notable exceptions (Embuscado *et al*, 2005; Yachida *et al*, 2010), the majority of these studies have been performed on therapy‐resistant lesions and might therefore not fully recapitulate the evolutionary processes occurring in drug‐naive cells (Dagogo‐Jack & Shaw, 2018; Duregon *et al*, 2019; Krook *et al*, 2019b). Moreover, the execution of such studies requires interdisciplinary collaboration across different medical and scientific disciplines, which can be difficult to be put in place, and necessitate the consent and close involvement of cancer patients and their families.

**Blood‐based** **tests**

High frequency monitoring of tumour evolution, especially over the course of therapeutic intervention, is required for the precise understanding of the mechanisms leading to drug resistance. Shedding light on this dynamic process could provide direct evidence of clonal selection and readily warn about the emergence of drug‐resistant clones. Evidently, these types of information could not be obtained through longitudinal multiregional biopsies, both for practical and ethical reasons, nor through rapid research autopsies. In recent years increasing attention has therefore been directed at detecting cancer‐derived components, such as circulating tumour cells (CTCs), as well as tumour‐derived components such as circulating tumour DNA (ctDNA), cell‐free RNAs, extracellular vesicles (EVs), proteins and metabolites (Ramalingam & Jeffrey, 2018; Rossi & Ignatiadis, 2019; Ignatiadis *et al*, 2021), in patients' bodily fluids. Provided that tumour cells and their components detected in the circulation represent their clonal frequency, blood‐based tests could allow the detection of changes occurring within a patient’s tumour in real time. As such, blood‐based tests have the potential to revolutionise personalised medicine and guide clinical decision‐making through constant assessment of ITH and tumour evolutionary dynamics via non‐invasive and cost‐effective monitoring of tumour growth, minimal residual disease (MRD), metastatisation and response to therapy. Growing evidence supports the feasibility of blood‐based tests to monitor tumour evolution across several tumour types (Misale *et al*, 2012; Dawson *et al*, 2013; Aceto *et al*, 2014; Scher *et al*, 2015; Siravegna *et al*, 2015; Abbosh *et al*, 2017, 2018; Anagnostou *et al*, 2019; Vidal *et al*, 2020). Additionally, blood‐based tests were shown to have prognostic value at the time of primary diagnosis and surgery in various solid tumours including breast, bladder, colorectal, head and neck and testicular cancer (Pantel & Alix‐Panabières, 2019; Chen *et al*, 2020) as well as melanoma (Lee *et al*, 2019) and NSCLC (Abbosh *et al*, 2017).

## Conflict of interest

K.L. has a patent on indel burden and CPI response pending and speaker fees from Roche tissue diagnostics, research funding from CRUK TDL/Ono/LifeArc alliance, and a consulting role with Monopteros Therapeutics. C.S. acknowledges grant support from Pfizer, AstraZeneca, Bristol Myers Squibb, Roche‐Ventana, Boehringer‐Ingelheim, Archer Dx Inc (collaboration in minimal residual disease sequencing technologies) and Ono Pharmaceuticals, is an AstraZeneca Advisory Board member and Chief Investigator for the MeRmaiD1 clinical trial. C.S. has consulted for Amgen, AstraZeneca, Bicycle Therapeutics, Bristol Myers Squibb, Celgene, Genentech, GlaxoSmithKline, GRAIL, Illumina, Medixci, Metabomed, MSD, Novartis, Pfizer, Roche‐Ventana and Sarah Cannon Research Institute. C.S. has stock options in Apogen Biotechnologies, Epic Biosciences, GRAIL, and has stock options and is co‐founder of Achilles Therapeutics. C.S. holds patents relating to assay technology to detect tumour recurrence (PCT/GB2017/ 053289); to targeting neoantigens (PCT/EP2016/059401); identifying patent response to immune checkpoint blockade (PCT/EP2016/071471); determining whether HLA LOH is lost in a tumour (PCT/GB2018/052004); predicting survival rates of cancer patients (PCT/GB2020/050221); to treating cancer by targeting insertion/deletion mutations (PCT/GB2018/051893); identifying insertion/deletion mutation targets (PCT/GB2018/051892); methods for lung cancer detection (PCT/US2017/028013); and identifying responders to cancer treatment (PCT/GB2018/051912).

## References

[embj2021108389-bib-0001] Abbosh C, Birkbak NJ, Wilson GA, Jamal‐Hanjani M, Constantin T, Salari R, Le Quesne J, Moore DA, Veeriah S, Rosenthal R*et al* (2017) Phylogenetic ctDNA analysis depicts early‐stage lung cancer evolution. Nature 545: 446–451 28445469 10.1038/nature22364 PMC5812436

[embj2021108389-bib-0002] Abbosh C, Birkbak NJ, Swanton C (2018) Early stage NSCLC — challenges to implementing ctDNA‐based screening and MRD detection. Nat Rev Clin Oncol 15: 577–586 29968853 10.1038/s41571-018-0058-3

[embj2021108389-bib-0003] Aceto N, Bardia A, Miyamoto D, Donaldson M, Wittner B, Spencer J, Yu M, Pely A, Engstrom A, Zhu H*et al* (2014) Circulating tumor cell clusters are oligoclonal precursors of breast cancer metastasis. Cell 158: 1110–1122 25171411 10.1016/j.cell.2014.07.013 PMC4149753

[embj2021108389-bib-0004] Aitken SJ, Anderson CJ, Connor F, Pich O, Sundaram V, Feig C, Rayner TF, Lukk M, Aitken S, Luft J*et al* (2020) Pervasive lesion segregation shapes cancer genome evolution. Nature 583: 265–270 32581361 10.1038/s41586-020-2435-1 PMC7116693

[embj2021108389-bib-0005] Albain K, Anderson S, Arriagada R, Barlow W, Bergh J, Bliss J, Buyse M, Cameron D, Carrasco E, Clarke M*et al* (2012) Comparisons between different polychemotherapy regimens for early breast cancer: meta‐analyses of long‐term outcome among 100 000 women in 123 randomised trials. Lancet 379: 432–444 22152853 10.1016/S0140-6736(11)61625-5 PMC3273723

[embj2021108389-bib-0006] Algazi AP, Othus M, Daud AI, Lo RS, Mehnert JM, Truong T‐G, Conry R, Kendra K, Doolittle GC, Clark JI*et al* (2020) Continuous versus intermittent BRAF and MEK inhibition in patients with BRAF‐mutated melanoma: a randomized phase 2 trial. Nat Med 26: 1564–1568 33020646 10.1038/s41591-020-1060-8 PMC8063889

[embj2021108389-bib-0007] Almendro V, Cheng Y‐K, Randles A, Itzkovitz S, Marusyk A, Ametller E, Gonzalez‐Farre X, Muñoz M, Russnes H, Helland Å*et al* (2014) Inference of tumor evolution during chemotherapy by computational modeling and in situ analysis of genetic and phenotypic cellular diversity. Cell Rep 6: 514–527 24462293 10.1016/j.celrep.2013.12.041 PMC3928845

[embj2021108389-bib-0008] Anagnostou V, Forde PM, White JR, Niknafs N, Hruban C, Naidoo J, Marrone K, Sivakumar IKA, Bruhm DC, Rosner S*et al* (2019) Dynamics of tumor and immune responses during immune checkpoint blockade in non–small cell lung cancer. Cancer Res 79: 1214–1225 30541742 10.1158/0008-5472.CAN-18-1127 PMC6432636

[embj2021108389-bib-0009] Anderson K, Lutz C, van Delft FW, Bateman CM, Guo Y, Colman SM, Kempski H, Moorman AV, Titley I, Swansbury J*et al* (2011) Genetic variegation of clonal architecture and propagating cells in leukaemia. Nature 469: 356–361 21160474 10.1038/nature09650

[embj2021108389-bib-0010] Ando K, Ohmori T, Inoue F, Kadofuku T, Hosaka T, Ishida H, Shirai T, Okuda K, Hirose T, Horichi N*et al* (2005) Enhancement of sensitivity to tumor necrosis factor α in non‐small cell lung cancer cells with acquired resistance to gefitinib. Clin Cancer Res 11: 8872–8879 16361577 10.1158/1078-0432.CCR-05-0811

[embj2021108389-bib-0011] André T, Boni C, Navarro M, Tabernero J, Hickish T, Topham C, Bonetti A, Clingan P, Bridgewater J, Rivera F*et al* (2009) Improved overall survival with oxaliplatin, fluorouracil, and leucovorin as adjuvant treatment in stage II or III colon cancer in the MOSAIC trial. J Clin Oncol 27: 3109–3116 19451431 10.1200/JCO.2008.20.6771

[embj2021108389-bib-0012] Aryee Mj, Liu W, Engelmann Jc, Nuhn P, Gurel M, Haffner Mc, Esopi D, Irizarry Ra, Getzenberg Rh, Nelson Wg*et al* (2013) DNA methylation alterations exhibit intraindividual stability and interindividual heterogeneity in prostate cancer metastases. Sci Transl Med 5: 169ra10 10.1126/scitranslmed.3005211 PMC3577373 23345608

[embj2021108389-bib-0013] Ascierto ML, Makohon‐Moore A, Lipson EJ, Taube JM, McMiller TL, Berger AE, Fan J, Kaunitz GJ, Cottrell TR, Kohutek ZA*et al* (2017) Transcriptional mechanisms of resistance to anti‐PD‐1 therapy. Clin Cancer Res 23: 3168–3180 28193624 10.1158/1078-0432.CCR-17-0270 PMC5474192

[embj2021108389-bib-0014] Avigdor BE, Cimino‐Mathews A, DeMarzo AM, Hicks JL, Shin J, Sukumar S, Fetting J, Argani P, Park BH, Wheelan SJ (2017) Mutational profiles of breast cancer metastases from a rapid autopsy series reveal multiple evolutionary trajectories. JCI Insight 2: e96896 10.1172/jci.insight.96896 PMC5752302 29263308

[embj2021108389-bib-0015] Azad TD, Chaudhuri AA, Fang P, Qiao Y, Esfahani MS, Chabon JJ, Hamilton EG, Yang YD, Lovejoy A, Newman AM*et al* (2020) Circulating tumor DNA analysis for detection of minimal residual disease after chemoradiotherapy for localized esophageal cancer. Gastroenterology 158: 494–505.e6 31711920 10.1053/j.gastro.2019.10.039 PMC7010551

[embj2021108389-bib-0016] Baca S, Prandi D, Lawrence M, Mosquera J, Romanel A, Drier Y, Park K, Kitabayashi N, MacDonald T, Ghandi M*et al* (2013) Punctuated evolution of prostate cancer genomes. Cell 153: 666–677 23622249 10.1016/j.cell.2013.03.021 PMC3690918

[embj2021108389-bib-0017] Bacevic K, Noble R, Soffar A, Wael Ammar O, Boszonyik B, Prieto S, Vincent C, Hochberg ME, Krasinska L, Fisher D (2017) Spatial competition constrains resistance to targeted cancer therapy. Nat Commun 8: 1995 29222471 10.1038/s41467-017-01516-1 PMC5722825

[embj2021108389-bib-0018] Bailey C, Shoura MJ, Mischel PS, Swanton C (2020) Extrachromosomal DNA—relieving heredity constraints, accelerating tumour evolution. Ann Oncol 31: 884–893 32275948 10.1016/j.annonc.2020.03.303

[embj2021108389-bib-0019] Bakhoum SF, Landau DA (2017) Chromosomal instability as a driver of tumor heterogeneity and evolution. Cold Spring Harb Perspect Med 7: a029611 28213433 10.1101/cshperspect.a029611 PMC5453382

[embj2021108389-bib-0020] Barthel FP, Johnson KC, Varn FS, Moskalik AD, Tanner G, Kocakavuk E, Anderson KJ, Abiola O, Aldape K, Alfaro KD*et al* (2019) Longitudinal molecular trajectories of diffuse glioma in adults. Nature 576: 112–120 31748746 10.1038/s41586-019-1775-1 PMC6897368

[embj2021108389-bib-0021] Bedard PL, Hyman DM, Davids MS, Siu LL (2020) Small molecules, big impact: 20 years of targeted therapy in oncology. Lancet 395: 1078–1088 32222192 10.1016/S0140-6736(20)30164-1

[embj2021108389-bib-0022] Bielski CM, Zehir A, Penson AV, Donoghue MTA, Chatila W, Armenia J, Chang MT, Schram AM, Jonsson P, Bandlamudi C*et al* (2018) Genome doubling shapes the evolution and prognosis of advanced cancers. Nat Genet 50: 1189–1195 30013179 10.1038/s41588-018-0165-1 PMC6072608

[embj2021108389-bib-0023] Boumahdi S, de Sauvage FJ (2020) The great escape: tumour cell plasticity in resistance to targeted therapy. Nat Rev Drug Discov 19: 39–56 31601994 10.1038/s41573-019-0044-1

[embj2021108389-bib-0024] Boveri T (2008) Concerning the origin of malignant tumours by Theodor Boveri. Translated and annotated by Henry Harris. J Cell Sci 121: 1–84 18089652 10.1242/jcs.025742

[embj2021108389-bib-0025] Bozic I, Paterson C, Waclaw B (2019) On measuring selection in cancer from subclonal mutation frequencies. PLoS Comput Biol 15: e1007368 31557163 10.1371/journal.pcbi.1007368 PMC6788714

[embj2021108389-bib-0251] Brabletz S, Schuhwerk H, Brabletz T, Stemmler MP (2021) Dynamic EMT: a multi‐tool for tumor progression. EMBO J 10.15252/embj.2021108647 PMC8441439 34459003

[embj2021108389-bib-0026] Brown D, Smeets D, Székely B, Larsimont D, Marcell Szász A, Adnet PY, Rothé F, Rouas G, Nagy ZI, Faragó Z*et al* (2017) Phylogenetic analysis of metastatic progression in breast cancer using somatic mutations and copy number aberrations. Nat Commun 8: 14944 28429735 10.1038/ncomms14944 PMC5474888

[embj2021108389-bib-0027] Brown TM, Fee E (2014) Rudolf Carl Virchow. Acta Gastroenterol Latinoam 44: 202 26742287

[embj2021108389-bib-0028] de Bruin EC, McGranahan N, Mitter R, Salm M, Wedge Dc, Yates L, Jamal‐Hanjani M, Shafi S, Murugaesu N, Rowan Aj*et al* (2014) Spatial and temporal diversity in genomic instability processes defines lung cancer evolution. Science 346: 251–256 25301630 10.1126/science.1253462 PMC4636050

[embj2021108389-bib-0029] Burrell RA, McClelland SE, Endesfelder D, Groth P, Weller M‐C, Shaikh N, Domingo E, Kanu N, Dewhurst SM, Gronroos E*et al* (2013) Replication stress links structural and numerical cancer chromosomal instability. Nature 494: 492–496 23446422 10.1038/nature11935 PMC4636055

[embj2021108389-bib-0030] Burrell RA, Swanton C (2014) Tumour heterogeneity and the evolution of polyclonal drug resistance. Mol Oncol 8: 1095–1111 25087573 10.1016/j.molonc.2014.06.005 PMC5528620

[embj2021108389-bib-0031] Caiado F, Silva‐Santos B, Norell H (2016) Intra‐tumour heterogeneity – going beyond genetics. FEBS J 283: 2245–2258 26945550 10.1111/febs.13705

[embj2021108389-bib-0032] Calabrese C, Davidson NR, Demircioğlu D, Fonseca NA, He Y, Kahles A, Lehmann K‐V, Liu F, Shiraishi Y, Soulette CM*et al* (2020) Genomic basis for RNA alterations in cancer. Nature 578: 129–136 32025019 10.1038/s41586-020-1970-0 PMC7054216

[embj2021108389-bib-0033] Campbell LL, Polyak K (2007) Breast tumor heterogeneity: cancer stem cells or clonal evolution? Cell Cycle 6: 2332–2338 17786053 10.4161/cc.6.19.4914

[embj2021108389-bib-0034] Campbell PJ, Yachida S, Mudie LJ, Stephens PJ, Pleasance ED, Stebbings LA, Morsberger LA, Latimer C, McLaren S, Lin M‐L*et al* (2010) The patterns and dynamics of genomic instability in metastatic pancreatic cancer. Nature 467: 1109–1113 20981101 10.1038/nature09460 PMC3137369

[embj2021108389-bib-0035] Cannataro VL, McKinley SA, St. Mary CM (2017) The evolutionary trade‐off between stem cell niche size, aging, and tumorigenesis. Evol Appl 10: 590–602 28616066 10.1111/eva.12476 PMC5469181

[embj2021108389-bib-0036] Chaudhuri AA, Chabon JJ, Lovejoy AF, Newman AM, Stehr H, Azad TD, Khodadoust MS, Esfahani MS, Liu CL, Zhou Li*et al* (2017) Early detection of molecular residual disease in localized lung cancer by circulating tumor DNA profiling. Cancer Discov 7: 1394–1403 28899864 10.1158/2159-8290.CD-17-0716 PMC5895851

[embj2021108389-bib-0037] Chen DY, Zhen HF, Qiu Y, Liu P, Zeng P, Xia J, Shi QY, Xie L, Zhu Z, Gao Y*et al* (2018) Comparison of single cell sequencing data between two whole genome amplification methods on two sequencing platforms. Sci Rep 8: 4963 29563514 10.1038/s41598-018-23325-2 PMC5862989

[embj2021108389-bib-0038] Chen HZ, Bonneville R, Roychowdhury S (2019) Implementing precision cancer medicine in the genomic era. Semin Cancer Biol 55: 16–27 29857039 10.1016/j.semcancer.2018.05.009

[embj2021108389-bib-0039] Chen X, Gole J, Gore A, He Q, Lu M, Min J, Yuan Z, Yang X, Jiang Y, Zhang T*et al* (2020) Non‐invasive early detection of cancer four years before conventional diagnosis using a blood test. Nat Commun 11: 3475 32694610 10.1038/s41467-020-17316-z PMC7374162

[embj2021108389-bib-0040] Chin RI, Chen K, Usmani A, Chua C, Harris PK, Binkley MS, Azad TD, Dudley JC, Chaudhuri AA (2019) Detection of solid tumor Molecular Residual Disease (MRD) using Circulating Tumor DNA (ctDNA). Mol Diagnosis Ther 23: 311–331 10.1007/s40291-019-00390-5 PMC6561896 30941670

[embj2021108389-bib-0041] Chkhaidze K, Heide T, Werner B, Williams MJ, Huang W, Caravagna G, Graham TA, Sottoriva A (2019) Spatially constrained tumour growth affects the patterns of clonal selection and neutral drift in cancer genomic data. PLoS Comput Biol 15: e1007243 31356595 10.1371/journal.pcbi.1007243 PMC6687187

[embj2021108389-bib-0042] Cibulskis K, Lawrence MS, Carter SL, Sivachenko A, Jaffe D, Sougnez C, Gabriel S, Meyerson M, Lander ES, Getz G (2013) Sensitive detection of somatic point mutations in impure and heterogeneous cancer samples. Nat Biotechnol 31: 213–219 23396013 10.1038/nbt.2514 PMC3833702

[embj2021108389-bib-0043] Clarke TL, Tang R, Chakraborty D, Van Rechem C, Ji F, Mishra S, Ma A, Kaniskan HÜ, Jin J, Lawrence MS*et al* (2020) Histone lysine methylation dynamics control EGFR DNA copy‐number amplification. Cancer Discov 10: 306–325 31776131 10.1158/2159-8290.CD-19-0463 PMC7271978

[embj2021108389-bib-0044] Cohen JD, Li Lu, Wang Y, Thoburn C, Afsari B, Danilova L, Douville C, Javed AA, Wong F, Mattox A*et al* (2018) Detection and localization of surgically resectable cancers with a multi‐analyte blood test. Science 359: 926–930 29348365 10.1126/science.aar3247 PMC6080308

[embj2021108389-bib-0045] Coombes RC, Page K, Salari R, Hastings RK, Armstrong A, Ahmed S, Ali S, Cleator S, Kenny L, Stebbing J*et al* (2019) Personalized detection of circulating tumor DNA antedates breast cancer metastatic recurrence. Clin Cancer Res 25: 4255–4263 30992300 10.1158/1078-0432.CCR-18-3663

[embj2021108389-bib-0046] Cottu PH, Asselah J, Lae M, Pierga JY, Diéras V, Mignot L, Sigal‐Zafrani B, Vincent‐Salomon A (2008) Intratumoral heterogeneity of HER2/neu expression and its consequences for the management of advanced breast cancer [2]. Ann Oncol 19: 596–597 32560012 10.1093/annonc/mdn021

[embj2021108389-bib-0047] Coussens LM, Werb Z (2002) Inflammation and cancer. Nature 420: 860–867 12490959 10.1038/nature01322 PMC2803035

[embj2021108389-bib-0048] Crook JM, O'Callaghan CJ, Duncan G, Dearnaley DP, Higano CS, Horwitz EM, Frymire E, Malone S, Chin J, Nabid A*et al* (2012) Intermittent androgen suppression for rising PSA level after radiotherapy. N Engl J Med 367: 895–903 22931259 10.1056/NEJMoa1201546 PMC3521033

[embj2021108389-bib-0049] Crosby D, Lyons N, Greenwood E, Harrison S, Hiom S, Moffat J, Quallo T, Samuel E, Walker I (2020) A roadmap for the early detection and diagnosis of cancer. Lancet Oncol 21: 1397–1399 33031732 10.1016/S1470-2045(20)30593-3 PMC7535618

[embj2021108389-bib-0050] Cross WCH, Graham TA, Wright NA (2016) New paradigms in clonal evolution: punctuated equilibrium in cancer. J Pathol 240: 126–136 27282810 10.1002/path.4757

[embj2021108389-bib-0051] Cunningham JJ (2019) A call for integrated metastatic management. Nat Ecol Evol 3: 996–998 31235925 10.1038/s41559-019-0927-x

[embj2021108389-bib-0052] Dagogo‐Jack I, Shaw AT (2018) Tumour heterogeneity and resistance to cancer therapies. Nat Rev Clin Oncol 15: 81–94 29115304 10.1038/nrclinonc.2017.166

[embj2021108389-bib-0053] Darwin CR (1859) On the origin of species by means of natural selection, or the preservation of favoured races in the struggle for life. London: John Murray. PMC5184128 30164232

[embj2021108389-bib-0054] Davis A, Gao R, Navin N (2017) Tumor evolution: Linear, branching, neutral or punctuated? Biochim Biophys Acta ‐ Rev Cancer 1867: 151–161 28110020 10.1016/j.bbcan.2017.01.003 PMC5558210

[embj2021108389-bib-0055] Dawson S‐J, Tsui DWY, Murtaza M, Biggs H, Rueda OM, Chin S‐F, Dunning MJ, Gale D, Forshew T, Mahler‐Araujo B*et al* (2013) Analysis of circulating tumor DNA to monitor metastatic breast cancer. N Engl J Med 368: 1199–1209 23484797 10.1056/NEJMoa1213261

[embj2021108389-bib-0056] de Koning HJ, van der Aalst CM, de Jong PA, Scholten ET, Nackaerts K, Heuvelmans MA, Lammers J‐W, Weenink C, Yousaf‐Khan U, Horeweg N*et al* (2020) Reduced lung‐cancer mortality with volume CT screening in a randomized trial. N Engl J Med 382: 503–513 31995683 10.1056/NEJMoa1911793

[embj2021108389-bib-0057] De Mattos‐Arruda L, Sammut S‐J, Ross EM, Bashford‐Rogers R, Greenstein E, Markus H, Morganella S, Teng Y, Maruvka Y, Pereira B*et al* (2019) The genomic and immune landscapes of lethal metastatic breast cancer. Cell Rep 27: 2690–2708.e10 31141692 10.1016/j.celrep.2019.04.098 PMC6546974

[embj2021108389-bib-0058] deCarvalho AC, Kim H, Poisson LM, Winn ME, Mueller C, Cherba D, Koeman J, Seth S, Protopopov A, Felicella M*et al* (2018) Discordant inheritance of chromosomal and extrachromosomal DNA elements contributes to dynamic disease evolution in glioblastoma. Nat Genet 50: 708–717 29686388 10.1038/s41588-018-0105-0 PMC5934307

[embj2021108389-bib-0059] DeGregori J, Pharoah P, Sasieni P, Swanton C (2020) Cancer screening, surrogates of survival, and the soma. Cancer Cell 38: 433–437 32946774 10.1016/j.ccell.2020.09.003

[embj2021108389-bib-0060] Deshpande V, Luebeck J, Nguyen NPD, Bakhtiari M, Turner KM, Schwab R, Carter H, Mischel PS, Bafna V (2019) Exploring the landscape of focal amplifications in cancer using AmpliconArchitect. Nat Commun 10: 392 30674876 10.1038/s41467-018-08200-y PMC6344493

[embj2021108389-bib-0061] Dewhurst SM, McGranahan N, Burrell RA, Rowan AJ, Grönroos E, Endesfelder D, Joshi T, Mouradov D, Gibbs P, Ward RL*et al* (2014) Tolerance of whole‐ genome doubling propagates chromosomal instability and accelerates cancer genome evolution. Cancer Discov 4: 175–185 24436049 10.1158/2159-8290.CD-13-0285 PMC4293454

[embj2021108389-bib-0062] Dexter DL, Kowalski HM, Blazar BA, Fligiel Z, Vogel R, Gloria H, Heppner H (1978) Heterogeneity of tumor cells from a single mouse mammary tumor. Cancer Res 38: 3174–3181 210930

[embj2021108389-bib-0063] Dudley JC, Schroers‐Martin J, Lazzareschi DV, Shi WY, Chen SB, Esfahani MS, Trivedi D, Chabon JJ, Chaudhuri AA, Stehr H*et al* (2019) Detection and surveillance of bladder cancer using urine tumor DNA. Cancer Discov 9: 500–509 30578357 10.1158/2159-8290.CD-18-0825 PMC6467650

[embj2021108389-bib-0064] Duregon E, Schneider J, DeMarzo AM, Hooper JE (2019) Rapid research autopsy is a stealthy but growing contributor to cancer research. Cancer 125: 2915–2919 31090935 10.1002/cncr.32184 PMC6690796

[embj2021108389-bib-0065] Efferth T, Saeed MEM, Kadioglu O, Seo EJ, Shirooie S, Mbaveng AT, Nabavi SM, Kuete V (2020) Collateral sensitivity of natural products in drug‐resistant cancer cells. Biotechnol Adv 38: 107342 30708024 10.1016/j.biotechadv.2019.01.009

[embj2021108389-bib-0066] Embuscado EE, Laheru D, Ricci F, Yun KJ, Witzel SDB, Seigel A, Flickinger K, Hidalgo M, Bova GS, Iacobuzio‐Donahue CA (2005) Immortalizing the complexity of cancer metastasis: Genetic features of lethal metastatic pancreatic cancer obtained from rapid autopsy. Cancer Biol Ther 4: 548–554 15846069 10.4161/cbt.4.5.1663 PMC2771924

[embj2021108389-bib-0067] Endesfelder D, Burrell RA, Kanu N, McGranahan N, Howell M, Parker PJ, Downward J, Swanton C, Kschischo M (2014) Chromosomal instability selects gene copy‐number variants encoding core regulators of proliferation in ER+ Breast cancer. Cancer Res 74: 4853–4863 24970479 10.1158/0008-5472.CAN-13-2664 PMC4167338

[embj2021108389-bib-0068] Enriquez‐Navas PM, Kam Y, Das T, Hassan S, Silva A, Foroutan P, Ruiz E, Martinez G, Minton S, Gillies RJ*et al* (2016) Exploiting evolutionary principles to prolong tumor control in preclinical models of breast cancer. Sci Transl Med 8 327ra24 10.1126/scitranslmed.aad7842 PMC4962860 26912903

[embj2021108389-bib-0069] Faltas BM, Prandi D, Tagawa ST, Molina AM, Nanus DM, Sternberg C, Rosenberg J, Mosquera JM, Robinson B, Elemento O*et al* (2016) Clonal evolution of chemotherapy‐resistant urothelial carcinoma. Nat Genet 48: 1490–1499 27749842 10.1038/ng.3692 PMC5549141

[embj2021108389-bib-0070] Fan J, Lee H‐O, Lee S, Ryu D‐E, Lee S, Xue C, Kim SJ, Kim K, Barkas N, Park PJ*et al* (2018) Linking transcriptional and genetic tumor heterogeneity through allele analysis of single‐cell RNA‐seq data. Genome Res 28: 1217–1227 29898899 10.1101/gr.228080.117 PMC6071640

[embj2021108389-bib-0071] Fan Y, Mao R, Lv H, Xu J, Yan L, Liu Y, Shi M, Ji G, Yu Y, Bai J*et al* (2011) Frequency of double minute chromosomes and combined cytogenetic abnormalities and their characteristics. J Appl Genet 52: 53–59 21107781 10.1007/s13353-010-0007-z

[embj2021108389-bib-0072] ffrench‐Constant RH, Bass C (2017) Does resistance really carry a fitness cost? Curr Opin Insect Sci 21: 39–46 28822487 10.1016/j.cois.2017.04.011 PMC5972224

[embj2021108389-bib-0073] Fittall MW, Van Loo P (2019) Translating insights into tumor evolution to clinical practice: promises and challenges. Genome Med 11: 20 30925887 10.1186/s13073-019-0632-z PMC6440005

[embj2021108389-bib-0074] Gao R, Davis A, McDonald TO, Sei E, Shi X, Wang Y, Tsai P‐C, Casasent A, Waters J, Zhang H*et al* (2016) Punctuated copy number evolution and clonal stasis in triple‐negative breast cancer. Nat Genet 48: 1119–1130 27526321 10.1038/ng.3641 PMC5042845

[embj2021108389-bib-0075] Gao Y, Ni X, Guo H, Su Z, Ba Yi, Tong Z, Guo Z, Yao X, Chen X, Yin J*et al* (2017) Single‐cell sequencing deciphers a convergent evolution of copy number alterations from primary to circulating tumor cells. Genome Res 27: 1312–1322 28487279 10.1101/gr.216788.116 PMC5538548

[embj2021108389-bib-0076] Gatenby RA, Brown JS (2020) Integrating evolutionary dynamics into cancer therapy. Nat Rev Clin Oncol 17: 675–686 32699310 10.1038/s41571-020-0411-1

[embj2021108389-bib-0077] Gatenby RA, Silva AS, Gillies RJ, Frieden BR (2009) Adaptive therapy. Cancer Res 69: 4894–4903 19487300 10.1158/0008-5472.CAN-08-3658 PMC3728826

[embj2021108389-bib-0078] Gatenby R, Vincent T (2008) An evolutionary model for initiation, promotion, and progression in carcinogenesis. Int J Oncol 32: 729–737 18360700

[embj2021108389-bib-0079] Gerlinger M, Horswell S, Larkin J, Rowan AJ, Salm MP, Varela I, Fisher R, McGranahan N, Matthews N, Santos CR*et al* (2014a) Genomic architecture and evolution of clear cell renal cell carcinomas defined by multiregion sequencing. Nat Genet 46: 225–233 24487277 10.1038/ng.2891 PMC4636053

[embj2021108389-bib-0080] Gerlinger M, McGranahan N, Dewhurst SM, Burrell RA, Tomlinson I, Swanton C (2014b) Cancer: evolution within a lifetime. Annu Rev Genet 48: 215–236 25292359 10.1146/annurev-genet-120213-092314

[embj2021108389-bib-0081] Gerlinger M, Rowan AJ, Horswell S, Larkin J, Endesfelder D, Gronroos E, Martinez P, Matthews N, Stewart A, Tarpey P*et al* (2012) Intratumor heterogeneity and branched evolution revealed by multiregion sequencing. N Engl J Med 366: 883–892 22397650 10.1056/NEJMoa1113205 PMC4878653

[embj2021108389-bib-0082] Gerlinger M, Swanton C (2010) How Darwinian models inform therapeutic failure initiated by clonal heterogeneity in cancer medicine. Br J Cancer 103: 1139–1143 20877357 10.1038/sj.bjc.6605912 PMC2967073

[embj2021108389-bib-0083] Gerstung M, Jolly C, Leshchiner I, Dentro SC, Gonzalez S, Rosebrock D, Mitchell TJ, Rubanova Y, Anur P, Yu K*et al* (2020) The evolutionary history of 2,658 cancers. Nature 578: 122–128 32025013 10.1038/s41586-019-1907-7 PMC7054212

[embj2021108389-bib-0084] Gillies RJ, Verduzco D, Gatenby RA (2012) Evolutionary dynamics of carcinogenesis and why targeted therapy does not work. Nat Rev Cancer 12: 487–493 22695393 10.1038/nrc3298 PMC4122506

[embj2021108389-bib-0085] Gisselsson D, Pettersson L, Höglund M, Heidenblad M, Gorunova L, Wiegant J, Mertens F, Dal Cin P, Mitelman F, Mandahl N (2000) Chromosomal breakage‐fusion‐bridge events cause genetic intratumor heterogeneity. Proc Natl Acad Sci U S A 97: 5357–5362 10805796 10.1073/pnas.090013497 PMC25833

[embj2021108389-bib-0086] Goldschmidt R (1941) The material basis of evolution. Philos Sci 8: 394

[embj2021108389-bib-0087] Grasso CS, Wu Y‐M, Robinson DR, Cao X, Dhanasekaran SM, Khan AP, Quist MJ, Jing X, Lonigro RJ, Brenner JC*et al* (2012) The mutational landscape of lethal castration‐resistant prostate cancer. Nature 487: 239–243 22722839 10.1038/nature11125 PMC3396711

[embj2021108389-bib-0088] Greaves M, Maley CC (2012) Clonal evolution in cancer. Nature 481: 306–313 22258609 10.1038/nature10762 PMC3367003

[embj2021108389-bib-0089] Gu X, Yu J, Chai P, Ge S, Fan X (2020) Novel insights into extrachromosomal DNA: redefining the onco‐drivers of tumor progression. J Exp Clin Cancer Res 39: 215 33046109 10.1186/s13046-020-01726-4 PMC7552444

[embj2021108389-bib-0090] Gundem G, Van Loo P, Kremeyer B, Alexandrov LB, Tubio JMC, Papaemmanuil E, Brewer DS, Kallio HML, Högnäs G, Annala M*et al* (2015) The evolutionary history of lethal metastatic prostate cancer. Nature 520: 353–357 25830880 10.1038/nature14347 PMC4413032

[embj2021108389-bib-0091] Gunnarsson EB, De S, Leder K, Foo J (2020) Understanding the role of phenotypic switching in cancer drug resistance. J Theor Biol 490: 110162 31953135 10.1016/j.jtbi.2020.110162 PMC7785289

[embj2021108389-bib-0092] Gutmann DH, Ferner RE, Listernick RH, Korf BR, Wolters PL, Johnson KJ (2017) Neurofibromatosis type 1. Nat Rev Dis Prim 3: 17004 28230061 10.1038/nrdp.2017.4

[embj2021108389-bib-0093] Haffner MC, De Marzo AM, Yegnasubramanian S, Epstein JI, Ballentine Carter H (2015) Diagnostic challenges of clonal heterogeneity in prostate cancer. J Clin Oncol 33: e38–e40 24638011 10.1200/JCO.2013.50.3540

[embj2021108389-bib-0094] Hata AN, Niederst MJ, Archibald HL, Gomez‐Caraballo M, Siddiqui FM, Mulvey HE, Maruvka YE, Ji F, Bhang H‐E, Krishnamurthy Radhakrishna V*et al* (2016) Tumor cells can follow distinct evolutionary paths to become resistant to epidermal growth factor receptor inhibition. Nat Med 22: 262–269 26828195 10.1038/nm.4040 PMC4900892

[embj2021108389-bib-0095] Hawkes N (2019) Cancer survival data emphasise importance of early diagnosis. BMJ 364: l408 30683652 10.1136/bmj.l408

[embj2021108389-bib-0096] Heppner GH (1984) Tumor heterogeneity. Cancer Res 44: 2259–2265 6372991

[embj2021108389-bib-0097] Hiley C, de Bruin EC, McGranahan N, Swanton C (2014) Deciphering intratumor heterogeneity and temporal acquisition of driver events to refine precision medicine. Genome Biol 15: 453 25222836 10.1186/s13059-014-0453-8 PMC4281956

[embj2021108389-bib-0098] Hirata E, Girotti MR, Viros A, Hooper S, Spencer‐Dene B, Matsuda M, Larkin J, Marais R, Sahai E (2015) Intravital imaging reveals how BRAF inhibition generates drug‐tolerant microenvironments with high integrin β1/FAK Signaling. Cancer Cell 27: 574–588 25873177 10.1016/j.ccell.2015.03.008 PMC4402404

[embj2021108389-bib-0099] Hoadley KA, Siegel MB, Kanchi KL, Miller CA, Ding Li, Zhao W, He X, Parker JS, Wendl MC, Fulton RS*et al* (2016) Tumor evolution in two patients with basal‐like breast cancer: a retrospective genomics study of multiple metastases. PLoS Medicine 13: e1002174 27923045 10.1371/journal.pmed.1002174 PMC5140046

[embj2021108389-bib-0100] Huminiecki L, Conant GC (2012) Polyploidy and the evolution of complex traits. Int J Evol Biol 2012: 1–12 10.1155/2012/292068 PMC3413983 22900230

[embj2021108389-bib-0101] Hussain M, Tangen CM, Berry DL, Higano CS, Crawford ED, Liu G, Wilding G, Prescott S, Kanaga Sundaram S, Small EJ*et al* (2013) Intermittent versus continuous androgen deprivation in prostate cancer. N Engl J Med 368: 1314–1325 23550669 10.1056/NEJMoa1212299 PMC3682658

[embj2021108389-bib-0102] Iacobuzio‐Donahue CA, Litchfield K, Swanton C (2020) Intratumor heterogeneity reflects clinical disease course. Nat Cancer 1: 3–6 10.1038/s43018-019-0002-1 35121835

[embj2021108389-bib-0103] Ignatiadis M, Sledge GW, Jeffrey SS (2021) Liquid biopsy enters the clinic — implementation issues and future challenges. Nat Rev Clin Oncol 18: 297–312 33473219 10.1038/s41571-020-00457-x

[embj2021108389-bib-0104] Jamal‐Hanjani M, Quezada SA, Larkin J, Swanton C (2015) Translational implications of tumor heterogeneity. Clin Cancer Res 21: 1258–1266 25770293 10.1158/1078-0432.CCR-14-1429 PMC4374162

[embj2021108389-bib-0105] Jamal‐Hanjani M, Wilson GA, McGranahan N, Birkbak NJ, Watkins TBK, Veeriah S, Shafi S, Johnson DH, Mitter R, Rosenthal R*et al* (2017) Tracking the evolution of non–small‐cell lung cancer. N Engl J Med 376: 2109–2121 28445112 10.1056/NEJMoa1616288

[embj2021108389-bib-0106] Joosse SA, Souche F‐R, Babayan A, Gasch C, Kerkhoven RM, Ramos J, Fabre J‐M, Riethdorf S, König A, Wikman H*et al* (2018) Chromosomal aberrations associated with sequential steps of the metastatic cascade in colorectal cancer patients. Clin Chem 64: 1505–1512 30030273 10.1373/clinchem.2018.289819

[embj2021108389-bib-0107] Kafle P, Peller P, Massolo A, Hoberg E, Leclerc LM, Tomaselli M, Kutz S (2020) Range expansion of muskox lungworms track rapid arctic warming: implications for geographic colonization under climate forcing. Sci Rep 10: 17323 33057173 10.1038/s41598-020-74358-5 PMC7560617

[embj2021108389-bib-0108] Kanu N, Cerone MA, Goh G, Zalmas L‐P, Bartkova J, Dietzen M, McGranahan N, Rogers R, Law EK, Gromova I*et al* (2016) DNA replication stress mediates APOBEC3 family mutagenesis in breast cancer. Genome Biol 17: 1–15 27634334 10.1186/s13059-016-1042-9 PMC5025597

[embj2021108389-bib-0109] Kemper K, De Goeje PL, Peeper DS, Van Amerongen R (2014) Phenotype switching: Tumor cell plasticity as a resistance mechanism and target for therapy. Cancer Res 74: 5937–5941 25320006 10.1158/0008-5472.CAN-14-1174

[embj2021108389-bib-0110] Kemper K, Krijgsman O, Cornelissen‐Steijger P, Shahrabi A, Weeber F, Song J‐Y, Kuilman T, Vis DJ, Wessels LF, Voest EE*et al* (2015) Intra‐ and inter‐tumor heterogeneity in a vemurafenib‐resistant melanoma patient and derived xenografts. EMBO Mol Med 7: 1104–1118 26105199 10.15252/emmm.201404914 PMC4568946

[embj2021108389-bib-0249] Kepp O, Bezu L, Yamazaki T, Di Virgilio F, Smyth MJ, Kroemer G, Galluzzi L (2021) ATP and cancer immunosurveillance. EMBO J 10.15252/embj.2021108130 PMC8246257 34121201

[embj2021108389-bib-0111] Khan KH, Cunningham D, Werner B, Vlachogiannis G, Spiteri I, Heide T, Mateos JF, Vatsiou A, Lampis A, Damavandi MD*et al* (2018) Longitudinal liquid biopsy and mathematical modeling of clonal evolution forecast time to treatment failure in the PROSPECT‐C Phase II colorectal cancer clinical trial. Cancer Discov 8: 1270–1285 30166348 10.1158/2159-8290.CD-17-0891 PMC6380469

[embj2021108389-bib-0112] Kim H, Nguyen N‐P, Turner K, Wu S, Gujar AD, Luebeck J, Liu J, Deshpande V, Rajkumar U, Namburi S*et al* (2020) Extrachromosomal DNA is associated with oncogene amplification and poor outcome across multiple cancers. Nat Genet 52: 891–897 32807987 10.1038/s41588-020-0678-2 PMC7484012

[embj2021108389-bib-0113] Kim IS, Heilmann S, Kansler ER, Zhang Y, Zimmer M, Ratnakumar K, Bowman RL, Simon‐Vermot T, Fennell M, Garippa R*et al* (2017) Microenvironment‐derived factors driving metastatic plasticity in melanoma. Nat Commun 8: 14343 28181494 10.1038/ncomms14343 PMC5309794

[embj2021108389-bib-0114] Kimura M (1983) The neutral theory of molecular evolution. Cambridge: Cambridge University Press

[embj2021108389-bib-0115] Kloosterman WP, Hoogstraat M, Paling O, Tavakoli‐Yaraki M, Renkens I, Vermaat JS, van Roosmalen MJ, van Lieshout S, Nijman IJ, Roessingh W*et al* (2011) Chromothripsis is a common mechanism driving genomic rearrangements in primary and metastatic colorectal cancer. Genome Biol 12: R103 22014273 10.1186/gb-2011-12-10-r103 PMC3333773

[embj2021108389-bib-0116] Koche RP, Rodriguez‐Fos E, Helmsauer K, Burkert M, MacArthur IC, Maag J, Chamorro R, Munoz‐Perez N, Puiggròs M, Dorado Garcia H*et al* (2020) Extrachromosomal circular DNA drives oncogenic genome remodeling in neuroblastoma. Nat Genet 52: 29–34 31844324 10.1038/s41588-019-0547-z PMC7008131

[embj2021108389-bib-0117] Kohl NE, Kanda N, Schreck RR, Bruns G, Latt SA, Gilbert F, Alt FW (1983) Transposition and amplification of oncogene‐related sequences in human neuroblastomas. Cell 35: 359–367 6197179 10.1016/0092-8674(83)90169-1

[embj2021108389-bib-0118] Korbel JO, Campbell PJ (2013) Criteria for inference of chromothripsis in cancer genomes. Cell 152: 1226–1236 23498933 10.1016/j.cell.2013.02.023

[embj2021108389-bib-0119] Krook MA, Bonneville R, Chen HZ, Reeser JW, Wing MR, Martin DM, Smith AM, Dao T, Samorodnitsky E, Paruchuri A*et al* (2019a) Tumor heterogeneity and acquired drug resistance in FGFR2‐fusion‐positive cholangiocarcinoma through rapid research autopsy. Cold Spring Harb Mol Case Stud 5: a004002 31371345 10.1101/mcs.a004002 PMC6672025

[embj2021108389-bib-0120] Krook MA, Chen HZ, Bonneville R, Allenby P, Roychowdhury S (2019b) Rapid research autopsy: piecing the puzzle of tumor heterogeneity. Trends Cancer 5: 1–5 30616752 10.1016/j.trecan.2018.11.004

[embj2021108389-bib-0121] Kutz SJ, Checkley S, Verocai GG, Dumond M, Hoberg EP, Peacock R, Wu JP, Orsel K, Seegers K, Warren AL*et al* (2013) Invasion, establishment, and range expansion of two parasitic nematodes in the canadian arctic. Glob Chang Biol 19: 3254–3262 23828740 10.1111/gcb.12315

[embj2021108389-bib-0122] La Manno G, Soldatov R, Zeisel A, Braun E, Hochgerner H, Petukhov V, Lidschreiber K, Kastriti ME, Lönnerberg P, Furlan A*et al* (2018) RNA velocity of single cells. Nature 560: 494–498 30089906 10.1038/s41586-018-0414-6 PMC6130801

[embj2021108389-bib-0123] Laconi E, Marongiu F, DeGregori J (2020) Cancer as a disease of old age: changing mutational and microenvironmental landscapes. Br J Cancer 122: 943–952 32042067 10.1038/s41416-019-0721-1 PMC7109142

[embj2021108389-bib-0124] Lasken RS (2013) Single‐cell sequencing in its prime. Nat Biotechnol 31: 211–212 23471069 10.1038/nbt.2523

[embj2021108389-bib-0125] Lee JH, Saw RP, Thompson JF, Lo S, Spillane AJ, Shannon KF, Stretch JR, Howle J, Menzies AM, Carlino MS*et al* (2019) Pre‐operative ctDNA predicts survival in high‐risk stage III cutaneous melanoma patients. Ann Oncol 30: 815–822 30860590 10.1093/annonc/mdz075 PMC6551453

[embj2021108389-bib-0126] Lin WW, Karin M (2007) A cytokine‐mediated link between innate immunity, inflammation, and cancer. J Clin Invest 117: 1175–1183 17476347 10.1172/JCI31537 PMC1857251

[embj2021108389-bib-0127] Ling S, Hu Z, Yang Z, Yang F, Li Y, Lin P, Chen Ke, Dong L, Cao L, Tao Y*et al* (2015) Extremely high genetic diversity in a single tumor points to prevalence of non‐Darwinian cell evolution. Proc Natl Acad Sci U S A 112: E6496–E6505 26561581 10.1073/pnas.1519556112 PMC4664355

[embj2021108389-bib-0128] Litchfield K, Stanislaw S, Spain L, Gallegos LL, Rowan A, Schnidrig D, Rosenbaum H, Harle A, Au L, Hill SM*et al* (2020) Representative sequencing: unbiased sampling of solid tumor tissue. Cell Rep 31: 107550 32375028 10.1016/j.celrep.2020.107550

[embj2021108389-bib-0129] Liu Mc, Oxnard Gr, Klein Ea, Swanton C, Seiden Mv, Liu MC, Oxnard GR, Klein EA, Smith D, Richards D*et al* (2020) Sensitive and specific multi‐cancer detection and localization using methylation signatures in cell‐free DNA. Ann Oncol 31: 745–759 33506766 10.1016/j.annonc.2020.02.011 PMC8274402

[embj2021108389-bib-0130] López S, Lim EL, Horswell S, Haase K, Huebner A, Dietzen M, Mourikis TP, Watkins TBK, Rowan A, Dewhurst SM*et al* (2020) Interplay between whole‐genome doubling and the accumulation of deleterious alterations in cancer evolution. Nat Genet 52: 283–293 32139907 10.1038/s41588-020-0584-7 PMC7116784

[embj2021108389-bib-0131] Lote H, Spiteri I, Ermini L, Vatsiou A, Roy A, McDonald A, Maka N, Balsitis M, Bose N, Simbolo M*et al* (2017) Carbon dating cancer: Defining the chronology of metastatic progression in colorectal cancer. Ann Oncol 28: 1243–1249 28327965 10.1093/annonc/mdx074 PMC5452067

[embj2021108389-bib-0132] Luria SE, Delbrück M (1943) Mutations of bacteria from virus sensitivity to virus resistance. Genetics 28: 491–511 17247100 10.1093/genetics/28.6.491 PMC1209226

[embj2021108389-bib-0133] Luskin MR, Murakami MA, Manalis SR, Weinstock DM (2018) Targeting minimal residual disease: a path to cure? Nat Rev Cancer 18: 255–263 29376520 10.1038/nrc.2017.125 PMC6398166

[embj2021108389-bib-0134] Machnik M, Oleksiewicz U (2020) Dynamic signatures of the epigenome: friend or foe? Cells 9: 653 10.3390/cells9030653 PMC7140607 32156057

[embj2021108389-bib-0135] Makohon‐Moore AP, Zhang M, Reiter JG, Bozic I, Allen B, Kundu D, Chatterjee K, Wong F, Jiao Y, Kohutek ZA*et al* (2017) Limited heterogeneity of known driver gene mutations among the metastases of individual patients with pancreatic cancer. Nat Genet 49: 358–366 28092682 10.1038/ng.3764 PMC5663439

[embj2021108389-bib-0136] Maley CC, Galipeau PC, Finley JC, Wongsurawat VJ, Li X, Sanchez CA, Paulson TG, Blount PL, Risques R‐A, Rabinovitch PS*et al* (2006) Genetic clonal diversity predicts progression to esophageal adenocarcinoma. Nat Genet 38: 468–473 16565718 10.1038/ng1768

[embj2021108389-bib-0137] Maley CC, Reid BJ, Forrest S (2004) Cancer prevention strategies that address the evolutionary dynamics of neoplastic cells: Simulating benign cell boosters and selection for chemosensitivity. Cancer Epidemiol Biomarkers Prev 13: 1375–1384 15298961

[embj2021108389-bib-0138] Malhotra A, Lindberg M, Faust GG, Leibowitz ML, Clark RA, Layer RM, Quinlan AR, Hall IM (2013) Breakpoint profiling of 64 cancer genomes reveals numerous complex rearrangements spawned by homology‐independent mechanisms. Genome Res 23: 762–776 23410887 10.1101/gr.143677.112 PMC3638133

[embj2021108389-bib-0139] Malikic S, Jahn K, Kuipers J, Sahinalp SC, Beerenwinkel N (2019) Integrative inference of subclonal tumour evolution from single‐cell and bulk sequencing data. Nat Commun 10: 2750 31227714 10.1038/s41467-019-10737-5 PMC6588593

[embj2021108389-bib-0140] Malkin D (2011) Li‐Fraumeni syndrome. Genes Cancer 2: 475–484 21779515 10.1177/1947601911413466 PMC3135649

[embj2021108389-bib-0141] Mallory XF, Edrisi M, Navin N, Nakhleh L (2020) Methods for copy number aberration detection from single‐cell DNA‐sequencing data. Genome Biol 21: 208 32807205 10.1186/s13059-020-02119-8 PMC7433197

[embj2021108389-bib-0142] Mardin BR, Drainas AP, Waszak SM, Weischenfeldt J, Isokane M, Stütz AM, Raeder B, Efthymiopoulos T, Buccitelli C, Segura‐Wang M*et al* (2015) A cell‐based model system links chromothripsis with hyperploidy. Mol Syst Biol 11: 828 26415501 10.15252/msb.20156505 PMC4592670

[embj2021108389-bib-0143] Marin‐Bejar O, Rogiers A, Dewaele M, Femel J, Karras P, Pozniak J, Bervoets G, Van Raemdonck N, Pedri D, Swings T*et al* (2021) Evolutionary predictability of genetic versus nongenetic resistance to anticancer drugs in melanoma. Cancer Cell 10.1016/j.ccell.2021.05.015 34143978

[embj2021108389-bib-0144] Marine JC, Dawson SJ, Dawson MA (2020) Non‐genetic mechanisms of therapeutic resistance in cancer. Nat Rev Cancer 20: 743–756 33033407 10.1038/s41568-020-00302-4

[embj2021108389-bib-0145] Martincorena I, Fowler JC, Wabik A, Lawson ARJ, Abascal F, Hall MWJ, Cagan A, Murai K, Mahbubani K, Stratton MR*et al* (2018) Somatic mutant clones colonize the human esophagus with age. Science 362: 911–917 30337457 10.1126/science.aau3879 PMC6298579

[embj2021108389-bib-0146] Martincorena I, Roshan A, Gerstung M, Ellis P, Van Loo P, McLaren S, Wedge DC, Fullam A, Alexandrov LB, Tubio JM*et al* (2015) High burden and pervasive positive selection of somatic mutations in normal human skin. Science 348: 880–886 25999502 10.1126/science.aaa6806 PMC4471149

[embj2021108389-bib-0147] Marusyk A, Tabassum DP, Altrock PM, Almendro V, Michor F, Polyak K (2014) Non‐cell‐autonomous driving of tumour growth supports sub‐clonal heterogeneity. Nature 514: 54–58 25079331 10.1038/nature13556 PMC4184961

[embj2021108389-bib-0148] Mayekar MK, Caswell DR, Vokes C‐DNI, Law E‐HEK, Wu W, Hill W, Gronroos E, Rowan A, Al Bakir M, Mccoach CE*et al* (2020) Targeted cancer therapy induces APOBEC fuelling the evolution of drug resistance. bioRxiv 10.1101/2020.12.18.423280 [PREPRINT]

[embj2021108389-bib-0149] McGranahan N, Burrell RA, Endesfelder D, Novelli MR, Swanton C (2012) Cancer chromosomal instability: therapeutic and diagnostic challenges. EMBO Rep 13: 528–538 22595889 10.1038/embor.2012.61 PMC3367245

[embj2021108389-bib-0150] McGranahan N, Swanton C (2015) Biological and therapeutic impact of intratumor heterogeneity in cancer evolution. Cancer Cell 27: 15–26 25584892 10.1016/j.ccell.2014.12.001

[embj2021108389-bib-0151] Merlo LMF, Pepper JW, Reid BJ, Maley CC (2006) Cancer as an evolutionary and ecological process. Nat Rev Cancer 6: 924–935 17109012 10.1038/nrc2013

[embj2021108389-bib-0152] Michor F, Iwasa Y, Nowak MA (2004) Dynamics of cancer progression. Nat Rev Cancer 4: 197–205 14993901 10.1038/nrc1295

[embj2021108389-bib-0252] Milan M, Diaferia GR, Natoli G (2021) Tumor cell heterogeneity and its transcriptional bases in pancreatic cancer: a tale of two cell types and their many variants. EMBO J 10.15252/embj.2020107206 PMC8246061 33844319

[embj2021108389-bib-0153] Mills JC, Stanger BZ, Sander M (2019) Nomenclature for cellular plasticity: are the terms as plastic as the cells themselves? EMBO J 38: e103148 31475380 10.15252/embj.2019103148 PMC6769377

[embj2021108389-bib-0154] Misale S, Yaeger R, Hobor S, Scala E, Janakiraman M, Liska D, Valtorta E, Schiavo R, Buscarino M, Siravegna G*et al* (2012) Emergence of KRAS mutations and acquired resistance to anti‐EGFR therapy in colorectal cancer. Nature 486: 532–536 22722830 10.1038/nature11156 PMC3927413

[embj2021108389-bib-0155] Mitchell TJ, Turajlic S, Rowan A, Nicol D, Farmery JHR, O’Brien T, Martincorena I, Tarpey P, Angelopoulos N, Yates LR*et al* (2018) Timing the landmark events in the evolution of clear cell renal cell cancer: TRACERx renal. Cell 173: 611–623.e17 29656891 10.1016/j.cell.2018.02.020 PMC5927631

[embj2021108389-bib-0156] Molenaar JJ, Koster J, Zwijnenburg DA, van Sluis P, Valentijn LJ, van der Ploeg I, Hamdi M, van Nes J, Westerman BA, van Arkel J*et al* (2012) Sequencing of neuroblastoma identifies chromothripsis and defects in neuritogenesis genes. Nature 483: 589–593 22367537 10.1038/nature10910

[embj2021108389-bib-0157] Morgan GJ (1998) Emile zuckerkandl, linus pauling, and the molecular evolutionary clock, 1959–1965. J Hist Biol 31: 155–178 11620303 10.1023/a:1004394418084

[embj2021108389-bib-0158] Mounier A, Georgiev D, Nam KN, Fitz NF, Castranio EL, Wolfe CM, Cronican AA, Schug J, Lefterov I, Koldamova R (2015) Bexarotene‐activated retinoid X receptors regulate neuronal differentiation and dendritic complexity. J Neurosci 35: 11862–11876 26311769 10.1523/JNEUROSCI.1001-15.2015 PMC4549399

[embj2021108389-bib-0159] Muller J (1973) On the nature and structural characteristics of cancer: general observations on the minute structure of morbid growths. CA Cancer J Clin 23: 307–312

[embj2021108389-bib-0160] Nam AS, Chaligne R, Landau DA (2021) Integrating genetic and non‐genetic determinants of cancer evolution by single‐cell multi‐omics. Nat Rev Genet 22: 3–18 32807900 10.1038/s41576-020-0265-5 PMC8450921

[embj2021108389-bib-0161] Nathanson DA, Gini B, Mottahedeh J, Visnyei K, Koga T, Gomez G, Eskin A, Hwang K, Wang J, Masui K*et al* (2014) Targeted therapy resistance mediated by dynamic regulation of extrachromosomal mutant EGFR DNA. Science 343: 72–76 24310612 10.1126/science.1241328 PMC4049335

[embj2021108389-bib-0162] Navin NE (2014) Cancer genomics: one cell at a time. Genome Biol 15: 452 25222669 10.1186/s13059-014-0452-9 PMC4281948

[embj2021108389-bib-0163] Navin NE (2015) The first five years of single‐cell cancer genomics and beyond. Genome Res 25: 1499–1507 26430160 10.1101/gr.191098.115 PMC4579335

[embj2021108389-bib-0164] Navin N, Kendall J, Troge J, Andrews P, Rodgers L, McIndoo J, Cook K, Stepansky A, Levy D, Esposito D*et al* (2011) Tumour evolution inferred by single‐cell sequencing. Nature 472: 90–95 21399628 10.1038/nature09807 PMC4504184

[embj2021108389-bib-0165] Navin N, Krasnitz A, Rodgers L, Cook K, Meth J, Kendall J, Riggs M, Eberling Y, Troge J, Grubor V*et al* (2010) Inferring tumor progression from genomic heterogeneity. Genome Res 20: 68–80 19903760 10.1101/gr.099622.109 PMC2798832

[embj2021108389-bib-0166] Naxerova K, Reiter JG, Brachtel E, Lennerz JK, van de Wetering M, Rowan A, Cai T, Clevers H, Swanton C, Nowak MA*et al* (2017) Origins of lymphatic and distant metastases in human colorectal cancer. Science 357: 357 10.1126/science.aai8515 PMC5536201 28684519

[embj2021108389-bib-0167] Nguyen DX, Bos PD, Massagué J (2009) Metastasis: from dissemination to organ‐specific colonization. Nat Rev Cancer 9: 274–284 19308067 10.1038/nrc2622

[embj2021108389-bib-0168] Nieto MA, Huang RYYJ, Jackson RAA, Thiery JPP (2016) Emt: 2016. Cell 166: 21–45 27368099 10.1016/j.cell.2016.06.028

[embj2021108389-bib-0169] Nik‐Zainal S, Alexandrov LB, Wedge DC, Van Loo P, Greenman CD, Raine K, Jones D, Hinton J, Marshall J, Stebbings LA*et al* (2012a) Mutational processes molding the genomes of 21 breast cancers. Cell 149: 979–993 22608084 10.1016/j.cell.2012.04.024 PMC3414841

[embj2021108389-bib-0170] Nik‐Zainal S, Van Loo P, Wedge DC, Alexandrov LB, Greenman CD, Lau KW, Raine K, Jones D, Marshall J, Ramakrishna M*et al* (2012b) The life history of 21 breast cancers. Cell 149: 994–1007 22608083 10.1016/j.cell.2012.04.023 PMC3428864

[embj2021108389-bib-0171] Notta F, Chan‐Seng‐Yue M, Lemire M, Li Y, Wilson GW, Connor AA, Denroche RE, Liang S‐B, Brown AMK, Kim JC*et al* (2016) A renewed model of pancreatic cancer evolution based on genomic rearrangement patterns. Nature 538: 378–382 27732578 10.1038/nature19823 PMC5446075

[embj2021108389-bib-0172] Nowell PC (1976) The clonal evolution of tumor cell populations. Science 194: 23–28 959840 10.1126/science.959840

[embj2021108389-bib-0173] Oobatake Y, Shimizu N (2020) Double‐strand breakage in the extrachromosomal double minutes triggers their aggregation in the nucleus, micronucleation, and morphological transformation. Genes Chromosom Cancer 59: 133–143 31569279 10.1002/gcc.22810

[embj2021108389-bib-0174] Pantel K, Alix‐Panabières C (2019) Liquid biopsy and minimal residual disease — latest advances and implications for cure. Nat Rev Clin Oncol 16: 409–424 30796368 10.1038/s41571-019-0187-3

[embj2021108389-bib-0250] Parker TM, Gupta K, Palma AM, Yekelchyk M, Fisher PB, Grossman SR, Won KJ, Madan E, Moreno E, Gogna R (2021) Cell competition in intratumoral and tumor microenvironment interactions. EMBO J 10.15252/embj.2020107271 PMC8408594 34368984

[embj2021108389-bib-0175] Patel AP, Tirosh I, Trombetta JJ, Shalek AK, Gillespie SM, Wakimoto H, Cahill DP, Nahed BV, Curry WT, Martuza RL*et al* (2014) Single‐cell RNA‐seq highlights intratumoral heterogeneity in primary glioblastoma. Science 344: 1396–1401 24925914 10.1126/science.1254257 PMC4123637

[embj2021108389-bib-0176] Pepper JW, Findlay CS, Kassen R, Spencer SL, Maley CC (2009) Cancer research meets evolutionary biology. Evol Appl 2: 62–70 25567847 10.1111/j.1752-4571.2008.00063.x PMC3352411

[embj2021108389-bib-0177] Pisapia DJ, Salvatore S, Pauli C, Hissong E, Eng K, Prandi D, Sailer V‐W, Robinson BD, Park K, Cyrta J*et al* (2017) Next‐generation rapid autopsies enable tumor evolution tracking and generation of preclinical models. JCO Precis Oncol 2017: 1–13 10.1200/PO.16.00038 PMC5761727 29333526

[embj2021108389-bib-0178] Pluchino KM, Hall MD, Goldsborough AS, Callaghan R, Gottesman MM (2012) Collateral sensitivity as a strategy against cancer multidrug resistance. Drug Resist Updat 15: 98–105 22483810 10.1016/j.drup.2012.03.002 PMC3348266

[embj2021108389-bib-0179] Pogrebniak KL, Curtis C (2018) Harnessing tumor evolution to circumvent resistance. Trends Genet 34: 639–651 29903534 10.1016/j.tig.2018.05.007 PMC6368975

[embj2021108389-bib-0248] Powles T, Assaf ZJ, Davarpanah N, Banchereau R, Szabados BE, Yuen KC, Grivas P, Hussain M, Oudard S, Gschwend JE*et al* (2021) ctDNA guiding adjuvant immunotherapy in urothelial carcinoma. Nature 595: 432–437 34135506 10.1038/s41586-021-03642-9

[embj2021108389-bib-0180] Pugh TJ, Delaney AD, Farnoud N, Flibotte S, Griffith M, Li HI, Qian H, Farinha P, Gascoyne RD, Marra MA (2008) Impact of whole genome amplification on analysis of copy number variants. Nucleic Acids Res 36: e80 18559357 10.1093/nar/gkn378 PMC2490749

[embj2021108389-bib-0181] Purushotham AD, Sullivan R (2010) Darwin, medicine and cancer. Ann Oncol 21: 199–203 19940013 10.1093/annonc/mdp537 PMC7135826

[embj2021108389-bib-0182] Ramalingam N, Jeffrey SS (2018) Future of liquid biopsies with growing technological and bioinformatics studies: opportunities and challenges in discovering tumor heterogeneity with single‐cell level analysis. Cancer J 24: 104–108 29601337 10.1097/PPO.0000000000000308 PMC5880298

[embj2021108389-bib-0183] Rambow F, Marine JC, Goding CR (2019) Melanoma plasticity and phenotypic diversity: therapeutic barriers and opportunities. Genes Dev 33: 1295–1318 31575676 10.1101/gad.329771.119 PMC6771388

[embj2021108389-bib-0184] Rambow F, Rogiers A, Marin‐Bejar O, Aibar S, Femel J, Dewaele M, Karras P, Brown D, Chang YH, Debiec‐Rychter M*et al* (2018) Toward minimal residual disease‐directed therapy in melanoma. Cell 174: 843–855.e19 30017245 10.1016/j.cell.2018.06.025

[embj2021108389-bib-0185] Ramón y Cajal S, Sesé M, Capdevila C, Aasen T, De Mattos‐Arruda L, Diaz‐Cano SJ, Hernández‐Losa J, Castellví J (2020) Clinical implications of intratumor heterogeneity: challenges and opportunities. J Mol Med 98: 161–177 31970428 10.1007/s00109-020-01874-2 PMC7007907

[embj2021108389-bib-0186] Razavi P, Chang MT, Xu G, Bandlamudi C, Ross DS, Vasan N, Cai Y, Bielski CM, Donoghue MTA, Jonsson P*et al* (2018) The genomic landscape of endocrine‐resistant advanced breast cancers. Cancer Cell 34: 427–438.e6 30205045 10.1016/j.ccell.2018.08.008 PMC6327853

[embj2021108389-bib-0187] Reiter JG, Baretti M, Gerold JM, Makohon‐Moore AP, Daud A, Iacobuzio‐Donahue CA, Azad NS, Kinzler KW, Nowak MA, Vogelstein B (2019) An analysis of genetic heterogeneity in untreated cancers. Nat Rev Cancer 19: 639–650 31455892 10.1038/s41568-019-0185-x PMC6816333

[embj2021108389-bib-0188] Reiter JG, Makohon‐Moore AP, Gerold JM, Heyde A, Attiyeh MA, Kohutek ZA, Tokheim CJ, Brown A, DeBlasio RM, Niyazov J*et al* (2018) Minimal functional driver gene heterogeneity among untreated metastases. Science 361: 1033–1037 30190408 10.1126/science.aat7171 PMC6329287

[embj2021108389-bib-0189] Riva L, Pandiri AR, Li YR, Droop A, Hewinson J, Quail MA, Iyer V, Shepherd R, Herbert RA, Campbell PJ*et al* (2020) The mutational signature profile of known and suspected human carcinogens in mice. Nat Genet 52: 1189–1197 32989322 10.1038/s41588-020-0692-4 PMC7610456

[embj2021108389-bib-0190] Rosenthal R, Cadieux EL, Salgado R, Bakir MA, Moore DA, Hiley CT, Lund T, Tanić M, Reading JL, Joshi K*et al* (2019) Neoantigen‐directed immune escape in lung cancer evolution. Nature 567: 479–485 30894752 10.1038/s41586-019-1032-7 PMC6954100

[embj2021108389-bib-0191] Rossi G, Ignatiadis M (2019) Promises and pitfalls of using liquid biopsy for precision medicine. Cancer Res 79: 2798–2804 31109952 10.1158/0008-5472.CAN-18-3402

[embj2021108389-bib-0192] Rozhok A, De Gregori J (2019) A generalized theory of age‐dependent carcinogenesis. Elife 8: e39950 31034356 10.7554/eLife.39950 PMC6488293

[embj2021108389-bib-0193] Russo M, Crisafulli G, Sogari A, Reilly NM, Arena S, Lamba S, Bartolini A, Amodio V, Magrì A, Novara L*et al* (2019) Adaptive mutability of colorectal cancers in response to targeted therapies. Science 366: 1473–1480 31699882 10.1126/science.aav4474

[embj2021108389-bib-0194] Salehi S, Steif A, Roth A, Aparicio S, Bouchard‐Côté A, Shah SP (2017) ddClone: joint statistical inference of clonal populations from single cell and bulk tumour sequencing data. Genome Biol 18: 44 28249593 10.1186/s13059-017-1169-3 PMC5333399

[embj2021108389-bib-0195] Sanchez‐Vega F, Hechtman JF, Castel P, Ku GY, Tuvy Y, Won H, Fong CJ, Bouvier N, Nanjangud GJ, Soong J*et al* (2019) Egfr and MET amplifications determine response to HER2 inhibition in ERBB2‐amplified esophagogastric cancer. Cancer Discov 9–2: 199–209 10.1158/2159-8290.CD-18-0598 PMC6368868 30463996

[embj2021108389-bib-0196] Scher HI, Heller G, Molina A, Attard G, Danila DC, Jia X, Peng W, Sandhu SK, Olmos D, Riisnaes R*et al* (2015) Circulating tumor cell biomarker panel as an individual‐level surrogate for survival in metastatic castration‐resistant prostate cancer. J Clin Oncol 33: 1348–1355 25800753 10.1200/JCO.2014.55.3487 PMC4397279

[embj2021108389-bib-0197] Schwartz R, Schäffer AA (2017) The evolution of tumour phylogenetics: principles and practice. Nat Rev Genet 18: 213–229 28190876 10.1038/nrg.2016.170 PMC5886015

[embj2021108389-bib-0198] Schweizer MT, Antonarakis ES, Wang H, Ajiboye AS, Spitz A, Cao H, Luo J, Haffner MC, Yegnasubramanian S, Carducci MA*et al* (2015) Effect of bipolar androgen therapy for asymptomatic men with castration‐resistant prostate cancer: results from a pilot clinical study. Sci Transl Med 7: 269ra2 10.1126/scitranslmed.3010563 PMC4507510 25568070

[embj2021108389-bib-0199] Shaffer SM, Dunagin MC, Torborg SR, Torre EA, Emert B, Krepler C, Beqiri M, Sproesser K, Brafford PA, Xiao M*et al* (2017) Rare cell variability and drug‐induced reprogramming as a mode of cancer drug resistance. Nature 546: 431–435 28607484 10.1038/nature22794 PMC5542814

[embj2021108389-bib-0200] Shah NP, Skaggs BJ, Branford S, Hughes TP, Nicoll JM, Paquette RL, Sawyers CL (2007) Sequential ABL kinase inhibitor therapy selects for compound drug‐resistant BCR‐ABL mutations with altered oncogenic potency. J Clin Invest 117: 2562–2569 17710227 10.1172/JCI30890 PMC1940237

[embj2021108389-bib-0201] Shah SP, Morin RD, Khattra J, Prentice L, Pugh T, Burleigh A, Delaney A, Gelmon K, Guliany R, Senz J*et al* (2009) Mutational evolution in a lobular breast tumour profiled at single nucleotide resolution. Nature 461: 809–813 19812674 10.1038/nature08489

[embj2021108389-bib-0202] Shapiro JR, Yung WKA, Shapiro WR, Shapiro JR, Shapiro WR (1981) Isolation, karyotype, and clonal growth of heterogeneous subpopulations of human malignant gliomas. Cancer Res 41: 2349–2359 7016313

[embj2021108389-bib-0203] Sharma SV, Lee DY, Li B, Quinlan MP, Takahashi F, Maheswaran S, McDermott U, Azizian N, Zou L, Fischbach MA*et al* (2010) A chromatin‐mediated reversible drug‐tolerant state in cancer cell subpopulations. Cell 141: 69–80 20371346 10.1016/j.cell.2010.02.027 PMC2851638

[embj2021108389-bib-0204] Shi H, Hugo W, Kong X, Hong A, Koya RC, Moriceau G, Chodon T, Guo R, Johnson DB, Dahlman KB*et al* (2014) Acquired resistance and clonal evolution in melanoma during BRAF inhibitor therapy. Cancer Discov 4: 80–93 24265155 10.1158/2159-8290.CD-13-0642 PMC3936420

[embj2021108389-bib-0205] Shimizu N, Itoh N, Utiyama H, Wahl GM (1998) Selective entrapment of extrachromosomally amplified DNA by nuclear budding and micronucleation during S phase. J Cell Biol 140: 1307–1320 9508765 10.1083/jcb.140.6.1307 PMC2132668

[embj2021108389-bib-0206] Siegel MB, He X, Hoadley KA, Hoyle A, Pearce JB, Garrett AL, Kumar S, Moylan VJ, Brady CM, Van Swearingen AED*et al* (2018a) Integrated RNA and DNA sequencing reveals early drivers of metastatic breast cancer. J Clin Invest 128: 1371–1383 29480819 10.1172/JCI96153 PMC5873890

[embj2021108389-bib-0207] Siegel RL, Miller KD, Jemal A (2017) Cancer statistics, 2017. CA Cancer J Clin 67: 7–30 28055103 10.3322/caac.21387

[embj2021108389-bib-0208] Siegel RL, Miller KD, Jemal A (2018b) Cancer statistics, 2018. CA Cancer J Clin 68: 7–30 29313949 10.3322/caac.21442

[embj2021108389-bib-0209] Simonsen AT, Hansen MC, Kjeldsen E, Møller PL, Hindkjær JJ, Hokland P, Aggerholm A (2018) Systematic evaluation of signal‐to‐noise ratio in variant detection from single cell genome multiple displacement amplification and exome sequencing. BMC Genom 19: 681 10.1186/s12864-018-5063-5 PMC6142419 30223769

[embj2021108389-bib-0210] Siravegna G, Mussolin B, Buscarino M, Corti G, Cassingena A, Crisafulli G, Ponzetti A, Cremolini C, Amatu A, Lauricella C*et al* (2015) Erratum: clonal evolution and resistance to EGFR blockade in the blood of colorectal cancer patients. Nat Med 21: 827 10.1038/nm0715-827b 26151329

[embj2021108389-bib-0211] Smith JC, Sheltzer JM (2018) Systematic identification of mutations and copy number alterations associated with cancer patient prognosis. Elife 7: e39217 30526857 10.7554/eLife.39217 PMC6289580

[embj2021108389-bib-0212] Smith MP, Brunton H, Rowling EJ, Ferguson J, Arozarena I, Miskolczi Z, Lee JL, Girotti MR, Marais R, Levesque MP*et al* (2016) Inhibiting drivers of non‐mutational drug tolerance is a salvage strategy for targeted melanoma therapy. Cancer Cell 29: 270–284 26977879 10.1016/j.ccell.2016.02.003 PMC4796027

[embj2021108389-bib-0213] Sottoriva A, Kang H, Ma Z, Graham TA, Salomon MP, Zhao J, Marjoram P, Siegmund K, Press MF, Shibata D*et al* (2015) A big bang model of human colorectal tumor growth. Nat Genet 47: 209–216 25665006 10.1038/ng.3214 PMC4575589

[embj2021108389-bib-0214] Stephens PJ, Greenman CD, Fu B, Yang F, Bignell GR, Mudie LJ, Pleasance ED, Lau KW, Beare D, Stebbings LA*et al* (2011) Massive genomic rearrangement acquired in a single catastrophic event during cancer development. Cell 144: 27–40 21215367 10.1016/j.cell.2010.11.055 PMC3065307

[embj2021108389-bib-0215] Storchova Z, Pellman D (2004) From polyploidy to aneuploidy, genome instability and cancer. Nat Rev Mol Cell Biol 5: 45–54 14708009 10.1038/nrm1276

[embj2021108389-bib-0216] Strobl MAR, West J, Viossat Y, Damaghi M, Robertson‐Tessi M, Brown JS, Gatenby RA, Maini PK, Anderson ARA (2021) Turnover modulates the need for a cost of resistance in adaptive therapy. Cancer Res 81: 1135–1147 33172930 10.1158/0008-5472.CAN-20-0806 PMC8455086

[embj2021108389-bib-0217] Swanton C (2012) Intratumor heterogeneity: evolution through space and time. Cancer Res 72: 4875–4882 23002210 10.1158/0008-5472.CAN-12-2217 PMC3712191

[embj2021108389-bib-0218] Takahashi T, Habuchi T, Kakehi Y, Mitsumori K, Akao T, Terachi T, Yoshida O (1998) Clonal and chronological genetic analysis of multifocal cancers of the bladder and upper urinary tract. Cancer Res 58: 5835–5841 9865743

[embj2021108389-bib-0219] Tarabichi M, Martincorena I, Gerstung M, Leroi AM, Markowetz F, Dentro SC, Leshchiner I, Jolly C, Haase K, Wintersinger J*et al* (2018) Neutral tumor evolution? Nat Genet 50: 1630–1633 30374075 10.1038/s41588-018-0258-x PMC6548558

[embj2021108389-bib-0220] Teixeira MR, Pandis N, Bardi G, Andersen JA, Heim S (1996) Karyotypic comparisons of multiple tumorous and macroscopically normal surrounding tissue samples from patients with breast cancer. Cancer Res 56: 855–859 8631024

[embj2021108389-bib-0221] Teixeira VH, Pipinikas CP, Pennycuick A, Lee‐Six H, Chandrasekharan D, Beane J, Morris TJ, Karpathakis A, Feber A, Breeze CE*et al* (2019) Deciphering the genomic, epigenomic, and transcriptomic landscapes of pre‐invasive lung cancer lesions. Nat Med 25: 517–525 30664780 10.1038/s41591-018-0323-0 PMC7614970

[embj2021108389-bib-0222] Tie J, Wang Y, Tomasetti C, Li L, Springer S, Kinde I, Silliman N, Tacey M, Wong HL, Christie M*et al* (2016) Circulating tumor DNA analysis detects minimal residual disease and predicts recurrence in patients with stage II colon cancer. Sci Transl Med 8: 346ra92 10.1126/scitranslmed.aaf6219 PMC5346159 27384348

[embj2021108389-bib-0223] Tung NM, Garber JE (2018) BRCA1/2 testing: therapeutic implications for breast cancer management. Br J Cancer 119: 141–152 29867226 10.1038/s41416-018-0127-5 PMC6048046

[embj2021108389-bib-0224] Turajlic S, Xu H, Litchfield K, Rowan A, Horswell S, Chambers T, O’Brien T, Lopez JI, Watkins TBK, Nicol D*et al* (2018) Deterministic evolutionary trajectories influence primary tumor growth: TRACERx Renal. Cell 173: 595–610.e11 29656894 10.1016/j.cell.2018.03.043 PMC5938372

[embj2021108389-bib-0225] Turner KM, Deshpande V, Beyter D, Koga T, Rusert J, Lee C, Li B, Arden K, Ren B, Nathanson DA*et al* (2017) Extrachromosomal oncogene amplification drives tumour evolution and genetic heterogeneity. Nature 543: 122–125 28178237 10.1038/nature21356 PMC5334176

[embj2021108389-bib-0226] Umbreit NT, Zhang C‐Z, Lynch LD, Blaine LJ, Cheng AM, Tourdot R, Sun L, Almubarak HF, Judge K, Mitchell TJ*et al* (2020) Mechanisms generating cancer genome complexity from a single cell division error. Science 368: eaba0712 32299917 10.1126/science.aba0712 PMC7347108

[embj2021108389-bib-0227] Van Loo P, Voet T (2014) Single cell analysis of cancer genomes. Curr Opin Genet Dev 24: 82–91 24531336 10.1016/j.gde.2013.12.004

[embj2021108389-bib-0228] Vendramin R, Katopodi V, Cinque S, Konnova A, Knezevic Z, Adnane S, Verheyden Y, Karras P, Demesmaeker E, Bosisio FM*et al* (2021) Activation of the integrated stress response confers vulnerability to mitoribosome‐targeting antibiotics in melanoma. J Exp Med 10.1084/jem.20210571 PMC8424468 34287642

[embj2021108389-bib-0229] Venkatesan S, Angelova M, Puttick C, Zhai H, Caswell DR, Lu W‐T, Dietzen M, Galanos P, Evangelou K, Bellelli R*et al* (2021) Induction of APOBEC3 exacerbates DNA replication stress and chromosomal instability in early breast and lung cancer evolution. Cancer Discov candisc.0725.2020. 10.1158/2159-8290.cd-20-0725 PMC8487921 33947663

[embj2021108389-bib-0230] Verhaak RGW, Bafna V, Mischel PS (2019) Extrachromosomal oncogene amplification in tumour pathogenesis and evolution. Nat Rev Cancer 19: 283–288 30872802 10.1038/s41568-019-0128-6 PMC7168519

[embj2021108389-bib-0231] Vidal J, Taus A, Montagut C (2020) Dynamic treatment stratification using ctDNA. Recent Results Cancer Res 215: 263–273 31605234 10.1007/978-3-030-26439-0_14

[embj2021108389-bib-0232] Waldman AD, Fritz JM, Lenardo MJ (2020) A guide to cancer immunotherapy: from T cell basic science to clinical practice. Nat Rev Immunol 20: 651–668 32433532 10.1038/s41577-020-0306-5 PMC7238960

[embj2021108389-bib-0233] Walter MJ, Shen D, Ding Li, Shao J, Koboldt DC, Chen K, Larson DE, McLellan MD, Dooling D, Abbott R*et al* (2012) Clonal architecture of secondary acute myeloid leukemia. N Engl J Med 366: 1090–1098 22417201 10.1056/NEJMoa1106968 PMC3320218

[embj2021108389-bib-0234] Wang Y, Waters J, Leung ML, Unruh A, Roh W, Shi X, Chen K, Scheet P, Vattathil S, Liang H*et al* (2014) Clonal evolution in breast cancer revealed by single nucleus genome sequencing. Nature 512: 155–160 25079324 10.1038/nature13600 PMC4158312

[embj2021108389-bib-0235] West JB, Dinh MN, Brown JS, Zhang J, Anderson AR, Gatenby RA (2019) Multidrug cancer therapy in metastatic castrate‐resistant prostate cancer: an evolution‐based strategy. Clin Cancer Res 25: 4413–4421 30992299 10.1158/1078-0432.CCR-19-0006 PMC6665681

[embj2021108389-bib-0236] Williams MJ, Werner B, Barnes CP, Graham TA, Sottoriva A (2016) Identification of neutral tumor evolution across cancer types. Nat Genet 48: 238–244 26780609 10.1038/ng.3489 PMC4934603

[embj2021108389-bib-0237] Winton T, Livingston R, Johnson D, Rigas J, Johnston M, Butts C, Cormier Y, Goss G, Inculet R, Vallieres E*et al* (2005) Vinorelbine plus cisplatin vs. observation in resected non–small‐cell lung cancer. N Engl J Med 352: 2589–2597 15972865 10.1056/NEJMoa043623

[embj2021108389-bib-0238] Wright NA (2014) Boveri at 100: cancer evolution, from preneoplasia to malignancy. J Pathol 234: 146–151 25043632 10.1002/path.4408

[embj2021108389-bib-0239] Wu CI, Wang HY, Ling S, Lu X (2016) The ecology and evolution of cancer: the ultra‐microevolutionary process. Annu Rev Genet 50: 347–369 27686281 10.1146/annurev-genet-112414-054842

[embj2021108389-bib-0240] Wu S, Turner KM, Nguyen N, Raviram R, Erb M, Santini J, Luebeck J, Rajkumar U, Diao Y, Li B*et al* (2019) Circular ecDNA promotes accessible chromatin and high oncogene expression. Nature 575: 699–703 31748743 10.1038/s41586-019-1763-5 PMC7094777

[embj2021108389-bib-0241] Wylie AA, Schoepfer J, Jahnke W, Cowan‐Jacob SW, Loo A, Furet P, Marzinzik AL, Pelle X, Donovan J, Zhu W*et al* (2017) The allosteric inhibitor ABL001 enables dual targeting of BCR‐ABL1. Nature 543: 733–737 28329763 10.1038/nature21702

[embj2021108389-bib-0242] Xie T, Musteanu M, Lopez‐Casas PP, Shields DJ, Olson P, Rejto PA, Hidalgo M (2015) Whole exome sequencing of rapid autopsy tumors and xenograft models reveals possible driver mutations underlying tumor progression. PLoS One 10: e0142631 26555578 10.1371/journal.pone.0142631 PMC4640827

[embj2021108389-bib-0243] Yachida S, Jones S, Bozic I, Antal T, Leary R, Fu B, Kamiyama M, Hruban RH, Eshleman JR, Nowak MA*et al* (2010) Distant metastasis occurs late during the genetic evolution of pancreatic cancer. Nature 467: 1114–1117 20981102 10.1038/nature09515 PMC3148940

[embj2021108389-bib-0244] Yizhak K, Aguet F, Kim J, Hess JM, Kübler K, Grimsby J, Frazer R, Zhang H, Haradhvala NJ, Rosebrock D*et al* (2019) RNA sequence analysis reveals macroscopic somatic clonal expansion across normal tissues. Science 364: eaaw0726 31171663 10.1126/science.aaw0726 PMC7350423

[embj2021108389-bib-0245] Yoshida K, Gowers KHC, Lee‐Six H, Chandrasekharan DP, Coorens T, Maughan EF, Beal K, Menzies A, Millar FR, Anderson E*et al* (2020) Tobacco smoking and somatic mutations in human bronchial epithelium. Nature 578: 266–272 31996850 10.1038/s41586-020-1961-1 PMC7021511

[embj2021108389-bib-0246] Zack TI, Schumacher SE, Carter SL, Cherniack AD, Saksena G, Tabak B, Lawrence MS, Zhang C‐Z, Wala J, Mermel CH*et al* (2013) Pan‐cancer patterns of somatic copy number alteration. Nat Genet 45: 1134–1140 24071852 10.1038/ng.2760 PMC3966983

[embj2021108389-bib-0247] Zhao B, Sedlak JC, Srinivas R, Creixell P, Pritchard JR, Tidor B, Lauffenburger DA, Hemann MT (2016) Exploiting temporal collateral sensitivity in tumor clonal evolution. Cell 165: 234–246 26924578 10.1016/j.cell.2016.01.045 PMC5152932

